# Persistence of activated anti‐mesothelin hYP218 chimeric antigen receptor T cells in the tumour is associated with efficacy in gastric and colorectal carcinomas

**DOI:** 10.1002/ctm2.70057

**Published:** 2024-11-15

**Authors:** Sameer Mir, Abhilash Venugopalan, Jingli Zhang, Nishanth Ulhas Nair, Manjistha Sengupta, Manakamana Khanal, Chaido Stathopoulou, Qun Jiang, Raffit Hassan

**Affiliations:** ^1^ Thoracic and GI Malignancies Branch, Center for Cancer Research (CCR), National Cancer Institute (NCI), National Institutes of Health (NIH) Bethesda Maryland USA; ^2^ Cancer Data Science Laboratory, Center for Cancer Research (CCR), National Cancer Institute (NCI), National Institutes of Health (NIH) Bethesda Maryland USA

**Keywords:** anti‐PD1, CAR T cell, colorectal cancer, gastric cancer, mesothelin, pembrolizumab, xenograft cancer model

## Abstract

Patients with advanced gastric and colorectal cancers have limited treatment options. Since mesothelin is highly expressed in these tumour types, we evaluated the therapeutic benefits of anti‐mesothelin hYP218 CAR T cells alone, and in combination with anti‐PD1 antibody, pembrolizumab.

GEPIA analysis was performed using human gastric (*n* = 408) and colon cancer tumours (*n* = 275) in TCGA database, to evaluate mRNA expression of mesothelin, compared to normal tissues. Mesothelin expression in gastric and colorectal cancer cell‐lines (*n* = 5) was analysed using flow cytometry. In vitro efficacy by hYP218 CAR T cells was tested by cytotoxicity and cytokine release assays. In vivo anti‐tumour efficacy of hYP218 CAR T cells alone, and in combination with pembrolizumab, was evaluated in NSG mice bearing human gastric (HGC27) and colorectal (SW48) tumour xenografts. Additionally, hYP218 CAR‐T cell persistence, activation and exhaustion marker‐expression were studied.

Mesothelin expression was significantly higher in gastric and colon cancer biopsies compared to normal tissues (*p* < .005). Mesothelin expression in gastric and colon cancer cell lines ranged from 10 000 to 70 000 molecules per cell. hYP218 CAR T cells demonstrated strong cytotoxic activity at low effector to target ratio, ranging from 0.24 to 1.0. In NSG mouse‐models, hYP218 CAR T cells demonstrated anti‐tumour efficacy and persisted in the tumour microenvironment in a functional state at day 40 posttreatment with expression of activation markers CD39 and CD69, increased production of IFN‐γ and TNF‐α and ability to kill tumour cells in vitro when isolated from tumours. There was increased PD1 expression. In combination with pembrolizumab, hYP218 CAR T cells led to slower tumour growth in NSG mice bearing large but not small HGC27 tumours. Anti‐tumour efficacy of hYP218 CAR T cells is due to increased accumulation of activated CAR T cells in the tumour and combination with pembrolizumab resulted in improvement in anti‐tumour activity of large established tumours.

**Highlights:**

- Mesothelin expression is significantly higher in gastric and colorectal cancers than normal tissues.
- hYP218 CAR T cells demonstrate strong anti‐tumour activity against mesothelin‐positive gastric and colorectal carcinomas.
- Activated hYP218 CAR T cells persist in the tumour microenvironment and retain their cytotoxic activity.
- Addition of pembrolizumab in larger tumours enhance CAR T cell efficacy.

## Graphical abstract

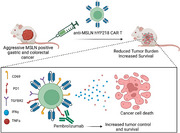

## 1 INTRODUCTION

Gastric cancer is one of the most prevalent cancers globally, and the third leading cause of cancer‐related deaths worldwide.^1^ Colorectal cancer is the third most frequently diagnosed cancer globally, exhibiting a higher prevalence, particularly in developed regions of the world.^2^, ^3^ Cumulatively, these malignancies account for a significant portion of morbidity and mortality, worldwide. The primary treatment modalities include surgery, chemotherapy, radiation therapy, and targeted therapies.^4^, ^5^, ^6^, ^7^ Despite therapeutic advances, most patients with locally advanced and metastatic disease have poor prognosis^3^, ^4^ and there is a need to develop better therapies.

Adoptive cell therapy using CAR T cells has shown remarkable clinical activity for treatment of hematologic malignancies, but its efficacy in treating solid tumours is limited,^8^, ^9^, ^10^, ^11^ including gastrointestinal cancers.^12^ One of the attractive cancer antigens for the treatment of solid tumours is mesothelin (MSLN), a cell surface glycoprotein that is highly expressed in many solid tumours but has limited expression on normal human tissues.^13^, ^14^ Several mesothelin‐targeted therapies including, immunotoxins,^15^, ^16^ monoclonal antibody,^17^ antibody drug conjugates,^18^ vaccine,^19^ and adoptive cell therapies,^20^, ^21^ have been evaluated in early‐phase clinical trials. A vast majority of these trials include patients with mesothelioma, ovarian and pancreatic cancer, where mesothelin expression has been well characterised.^22^ However, recent studies have shown that mesothelin is also expressed in a small subset of patients with other cancer types that account for a substantial fraction of cancer related deaths worldwide. For example, about a quarter of patients with lung adenocarcinoma express high levels of mesothelin, especially those with KRAS mutant lung cancer.^23^ Similarly, recent pathologic studies have shown that mesothelin is highly expressed in a subset of patients with gastric and colorectal carcinomas.^24^, ^25^, ^26^, ^27^ Our previous preclinical studies of anti‐mesothelin hYP218 CAR T cells targeting the membrane‐proximal epitope of mesothelin have shown promising results, including effective tumour cell eradication and increased survival in animal models involving various cancer types, such as mesothelioma, pancreatic, and ovarian cancers.^28^ In this study, we evaluated the potential of anti‐mesothelin hYP218 CAR T cells for the treatment of gastric and colorectal cancers and understand their phenotypic characteristics in the tumour. We demonstrate that hYP218 CAR‐T cell therapy led to a significant reduction in tumour growth and improved survival in gastric and colorectal tumour xenograft models. Notably, hYP218 CAR T cells were maintained in an activated state in the tumour microenvironment, indicated by the expression of CD39 and CD69 markers along with increased IFNγ, and TNFα secretion. Although hYP218 CAR T cells upregulated the PD1 molecule, treatment with anti‐PD1 inhibitor pembrolizumab produced only a modest antitumour response.

## 2 MATERIALS AND METHODS

### 2.1 GEPIA analysis for mesothelin expression in human gastric and colorectal cancers

GEPIA (Gene Expression Profiling Interactive Analysis) is a web server tool for gene expression studies (http://gepia.cancer‐pku.cn/). It utilises expression data from 9736 tumour and 8587 normal tissues from the TCGA (The Cancer Genome Atlas) and GTEx (Genotype‐Tissue Expression) repositories. Tumour types analysed for this study has been tabulated in Table S1. The Kaplan–Meier survival analysis was performed for subjects with high and low MSLN expressing tumours as determined by RNA‐seq data available on TCGA database utilising GEPIA. The median values between the lower (lowest 25%) and upper (top 25%) quartiles were automatically chosen to categorise them into high and low expression groups.

### 2.2 Mice, established tumour cell lines and healthy donor derived T cells

The Animal Care and Use Committee (ACUC) of the NCI, NIH (Bethesda, MD), granted approval for all animal experiments. NOD SCID gamma (NSG) mice were obtained from The Jackson Laboratory (Bar Harbor, ME, USA). All mice were housed in a pathogen‐free environment following NIH guidelines approved by the NCI's Animal Care and Use Committee. For the in vivo studies, 5‐ to 6‐week‐old NSG mice were used and housed under ambient conditions with unrestricted access to water and standard rodent chow. The well‐being of the animals was closely monitored, and they were euthanised upon reaching disease model‐specific endpoints to minimise suffering. Since the HGC27 and SW48 cell lines used for our in vivo studies are very aggressive, the mouse tumours were monitored for tumour growth twice a week and the mice were euthanised immediately if the tumour volume had reached more than 2000 mm^3^. Subcutaneous tumour xenograft experiments included groups with at least 5 mice each to ensure statistical relevance. The tumour cell lines N87, HGC27, H1703 (obtained from Dr. Ji Lou, Center for Cancer Research, NCI, NIH, Bethesda, MD), and AGS (obtained from ATCC), SW48 and HTB39 (obtained from Dr. Ira Pastan, Center for Cancer Research, NCI, NIH, Bethesda, MD), and OVCAR8 (received from Dr. Hisataka Kobayashi, Center for Cancer Research, NCI, NIH, Bethesda, MD) were maintained in RPMI1640 medium containing 10% FBS, 2 mmol/L L‐glutamine, and 100 U penicillin‐streptomycin. These cell lines were authenticated and routinely tested for the absence of Mycoplasma using short tandem repeat profiling.

PBMCs from healthy donors were provided by the NIH Clinical Center Department of Transfusion Medicine as part of their IRB approved and consented healthy donor program. PBMC isolation from the peripheral blood samples was performed using lymphocyte separation medium 1077 (Promocell, catalogue no. C‐44010).

### 2.3 Mesothelin expression in tumour cell lines

The cell surface expression of mesothelin was evaluated in the established gastric cancer cell lines N87, HGC27 and AGS; colorectal cancer cell lines SW48 and HTB39. OVCAR‐8 (ovarian cancer), and H1703 (lung cancer) were used as positive and negative controls, respectively, for cell surface mesothelin expression. Flow cytometry was used to determine the surface mesothelin expression of these tumour cell lines using the mouse MN antibody (Rockland Inc., catalogue no. 200‐301‐A88). 0.1‐0.3 million cells were stained with 0.5 µL of the anti‐MSLN antibody, incubated at 4°C for 30 min, and washed with PBS before acquisition. A standard curve was generated using PE quantibrite beads (BD Biosciences, catalogue no. 340495) to quantify mesothelin molecules per cell.

### 2.4 CAR plasmid design, lentivirus transduction, and CAR T cell expansion

The generation of hYP218 CAR T cells has been previously described. Briefly, it consists of a humanised rabbit anti‐mesothelin antibody (hYP218) that binds to the membrane proximal region of mesothelin, and a second‐generation CAR vector which was created by inserting the scFv sequences into the lentivirus plasmid backbone under the control of the EF1alpha promoter. These sequences included the hinge and transmembrane domains of CD8α, the 4‐1BB costimulatory domain, and the CD3ζ signalling domain. For in vitro and in vivo tracking, and CAR T‐cell ablation, if needed to reduce toxicity, a truncated human EGFR polypeptide (huEGFRt) was added. A T2A self‐cleaved sequence separates the CAR from the huEGFRt. VSVG pseudotyped lentivirus with a high titre was developed by Cellomics Technology (Halethorpe, MD). T cells were expanded ex vivo by activating PBMCs from healthy donors with anti‐CD3/CD28 Transact (Miltenyi Biotec, catalogue no. 130‐111‐160) in the presence of IL2, IL7, and IL15 (Peprotech, catalogue no's: 200–02, ‐07,‐15) in RPMI media. CAR lentivirus was transduced into cells at a multiplicity of infection (MOI) of 5 in the presence of protamine sulphate. On day 9 posttransduction, CAR T cells (or untransduced T cells) were used for in vitro and in vivo studies. Flow cytometry was used to determine the surface expression of human T cell markers, huEGFRt, and MSLN (Table S2).

### 2.5 In vitro cytotoxicity and cytokine assays for evaluating CAR‐T cell effector functions

The effector function of CAR T cells was evaluated in vitro by cytotoxicity assay, where killing of mesothelin‐expressing tumour cells on co‐culture with CAR T cells and cytokines secreted were measured. Cytotoxicity assays were performed by plating 5000 tumour cells in 100 µL of complete RPMI medium in a 96‐well plate. After seeding the tumour cells for 4–6 h, 50 µL of culture containing CAR T cells or untransduced T cells at various effector‐to‐target (E:T) ratios was added to initiate co‐culture. Wells containing only tumour cells, without CAR T cells, served as internal plate standards. After 20 to 24 h of co‐culture, the fluorescence signal from surviving tumour cells was measured using the CellTiter‐Glo® 2.0 Cell Viability Assay (Promega, catalogue no. G9243). The cytotoxic assay involving OVCAR8 was performed by using luciferase assay system (Promega, catalogue no. E1501). The percentage of cell killing was determined using the formula: % killing = 100 [1 ‐ relative fluorescence units (RFUs) from co‐culture wells/RFUs from target‐alone wells]. IL2, IFNγ, and TNFα levels secreted by stimulated CAR T cells after 20 to 24 h of co‐culture with tumour cells were quantified using ELISA kits (R&D Systems, catalogue No. D2050, DIF50C, DTA00D). Briefly, in a 96‐well plate, 5000 cancer cells were seeded per well and then co‐incubated with different ratios of hYP218 CAR T cells. After 24 h, the supernatant was collected for cytokine release assays.

### 2.6 Flow cytometry analysis of hYP218 CAR T cell activation and exhaustion

We used flow cytometry to evaluate the transduction efficiency of CAR T cells and determine their activation and exhaustion profiles. Posttransduction, CAR T cells were collected, washed, and stained with fluorescently labelled antibodies. For detecting CAR T expression, an antibody targeting the extracellular domain of huEGFRt was utilised. The activation status of the CAR T cells was determined using anti‐CD69 and anti‐CD39 antibodies. To assess the exhaustion profile, the cells were stained with antibodies against PD1, TIM3, and LAG3, three classical markers indicative of T cell exhaustion along with TGFBR2 (Table S2). Antibodies against hCD3 were used to gate the T cells, and UV Zombie UV™ dye (Biolegend, catalogue no. 77474) was used to gate the live cells. For intracellular staining of IFNγ, TNFα, and IL2, cells were incubated for 4 h with 2 µL/mL of Leukocyte Activation Cocktail (BD Biosciences, catalogue no. 550583) at 37°C in a tissue culture incubator. Surface antibodies were stained first followed by fixation, using Cytofix/Cytoperm (BD Biosciences, catalogue no. 554722), and intracellular cytokine staining. Poly‐ and mono‐cytokine producing hYP218 CAR T cells were analysed by Boolean gate analysis. At least 10 000 cells were analysed in the in vitro experiments, and 0.5 million cells were analysed in the in vivo experiments. All stained cells were subsequently washed to remove unbound antibodies and resuspended in appropriate flow cytometry buffer. Data acquisition was performed on a CytoFLEX LX flow cytometer (Beckman Coulter). Compensation and analysis of the acquired data were conducted using the BD software, FlowJo 10.8.1. Gating strategies were devised to delineate CAR‐expressing T cells, activated T cells, and T cells expressing the exhaustion markers.

### 2.7 Repeated tumour antigen stimulation of hYP218 CAR T cells

To evaluate the sustainability and responsiveness of the CAR T cells to repeated tumour antigen challenges, in vitro repeated tumour exposure was performed. HGC27 and SW48 cell lines that highly express mesothelin were used as target cells for chronic stimulation assay. hYP218 CAR T cells and target tumour cells were co‐cultured at an effector to target (E:T) ratio of 1:1 on day 0. hYP218 CAR T cells were subjected to repeated restimulation on days 1, 3, and 6. For each restimulation, the medium containing CAR T cells was carefully removed, and the percentage of CAR T cells present was assessed. Fresh tumour cells were then added to the culture to ensure continued engagement of the CAR T cells with their targets in the same E:T ratio. hYP218 CAR T cells were subjected to analysis at the beginning (day 0), middle (day 4) and end (day 7) of a week‐long assay to assess their effector functions as well as their activation and exhaustion status. The CFSE assay was used to assess the T cell proliferation. Cells were labelled with 5µM CellTrace CFSE dye (Invitrogen, catalogue no. C3455), and the dilution of fluorescence intensity was measured by flow cytometry to track cell division over time.

### 2.8 In vivo activity of hYP218 CAR T cells

The in vivo antitumour efficacy of CAR T cells was evaluated using two distinct human xenograft models: HGC27 (gastric cancer) and SW48 (colorectal cancer) tumour models. HGC27 and SW48 cell lines were selected for in vivo experiments owing to their high mesothelin expression, shorter doubling time, and ability to form robust tumours, making them ideal for our study requirements. Subcutaneous implantation of 1 × 10^6^ HGC27 or SW48 tumour cells into the right flank region of female NSG mice (*n* = 5/7) aged 5 to 6 weeks was performed. Randomisation and Standardised procedures and protocols for treatment administration and tumour volume measurements were used to minimise the potential confounders. The volume of subcutaneous tumours was calculated using the formula: tumour volume (mm^3^) = length × width^2^ × 0.4. When the tumour volume reached 80–100 mm^3^, a single intravenous injection of 5 × 10^6^ CAR T cells was administered. The control groups receiving saline and untransduced T respectively, serve as a comparison to assess the specific effects of h YP218 CAR T cells. The survival status of the mice was monitored in real‐time, and the volume of the tumours was measured twice a week. Peripheral blood from the mice was collected, allowed to clot at room temperature for 30 to 60 min, centrifuged at 3000 rpm for 15 min to separate the serum from the cellular components and carefully transferred to a microcentrifuge tube. Mechanical and Liberase (Roche, catalogue no. 05401020001) enzymatic digestion methods^29^ were used to prepare single cell suspensions from the spleen and tumours of the euthanised mice.

### 2.9 Persistence and characterisation of CAR T cells in the tumour and spleen

To evaluate the long‐term persistence of CAR T cells, blood, tumour, and spleen tissues were harvested from the mice for comprehensive analysis on day 40 posttreatment. This allowed us to evaluate the presence and functional state of the infused CAR T cells. Liberase TL enzyme, in conjunction with mechanical procedures, were used to produce single‐cell suspensions from the extracted tissues. Flow cytometry was employed to check for the expression of various immunological markers, including those indicating activation, exhaustion, and the presence of inhibitory molecules like PD1 and TGFBR2. For isolating tumour‐infiltrating lymphocytes, cells were first labelled with anti‐CD3 PE antibodies (Biolegend, catalogue no. 980008), followed by labelling with PE‐conjugated microbeads (Miltenyi, catalogue no.130‐048‐801). The cell suspension was then loaded onto an LS column (Miltenyi, catalogue no. 130‐042‐401) placed in a magnetic field, allowing the isolation of CD3+ TILs through positive selection.

### 2.10 Statistical analysis

Unpaired Student's *t*‐test was used to compare the differences between the untransduced and hYP218 CAR T cells in the in vitro cytotoxicity and cytokine release assay. Mouse survival differences were compared using log‐rank (Mantel‐Cox) test. Statistical analysis was performed using GraphPad Prism 9.0. A value of *p* < .05 was considered statistically significant.

## 3 RESULTS

### 3.1 Mesothelin expression in gastric and colorectal tumours and cell lines

**FIGURE 1:**
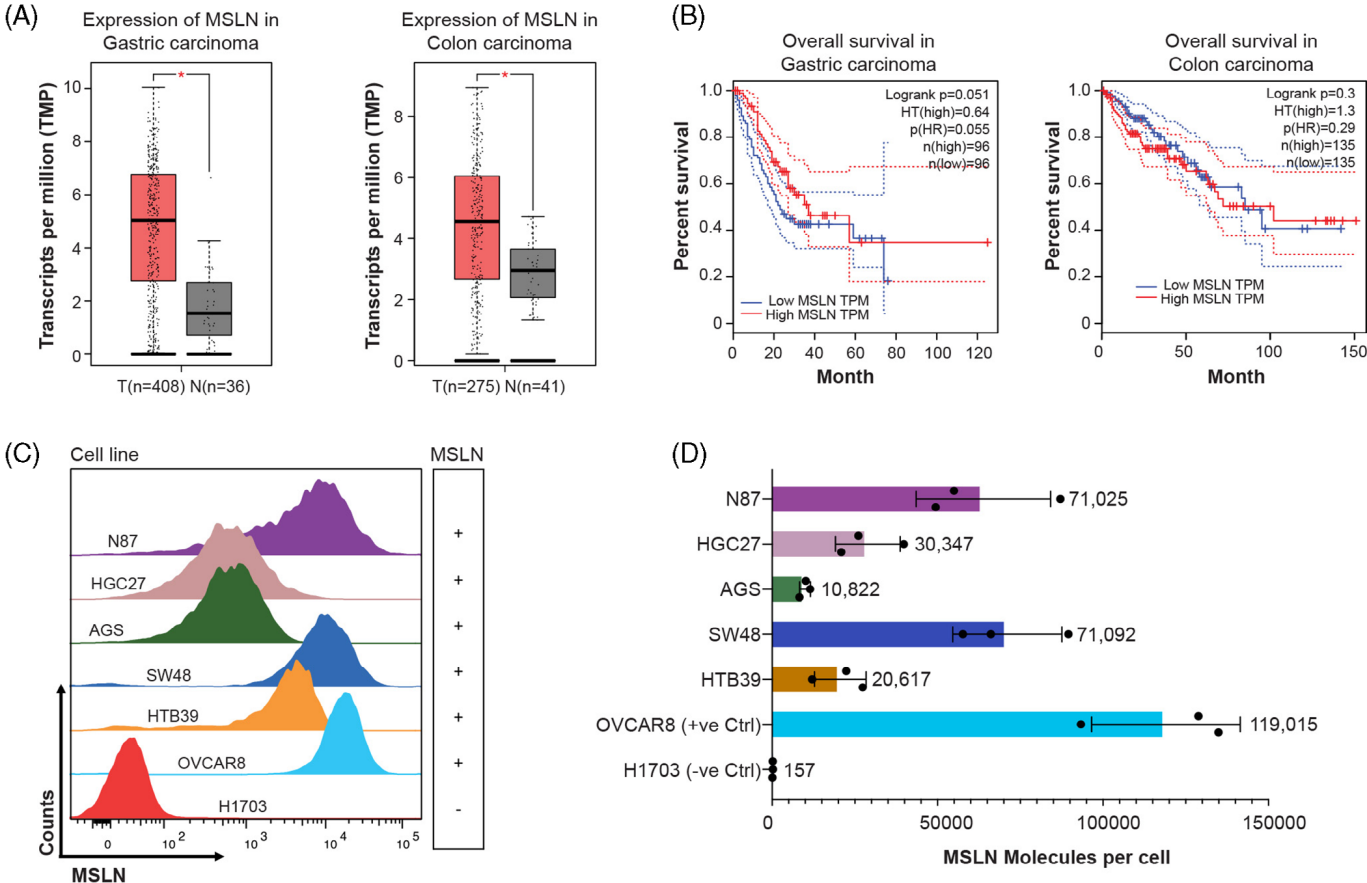
Mesothelin (MSLN) expression in human tumours and cell lines. (A) MSLN RNA expression in gastric and colon tumours (T) as compared to matched healthy tissue (N) in TCGA pan‐cancer data. For gastric cancer 408 tumours and 36 matched healthy tissues were analysed. For colon carcinoma 275 tumours and 41 matched healthy tissues were analysed. The Log2FC cutoff was set at 1, and the *p*‐value cutoff was set at 0.01. (B) Kaplan–Meyer plot showing overall survival in patients with high and low levels of tumour mesothelin expression in gastric (left) and colon carcinomas (right). The 95% confidence interval (CI) is represented as a dotted line. The group cutoff is selected based on quartiles, with top 25% and minimum 25% MSLN expression. (C) Histograms displaying MSLN cell surface expression by flow cytometry in various gastric (AGS, N87 and HCG27) and colorectal cancer cell lines (SW48, HTB39). Ovarian cancer cell line OVCAR8 and non‐small cell lung cancer cell line H1703 were used as positive and negative controls respectively for MSLN expression. (D) The quantification of MSLN molecules per cell was determined by flow cytometry using anti‐human mesothelin antibody conjugated with PE quantibrite beads. Gating strategy: FSC/SSC‐Singlets‐Live‐MSLN+. Data are plotted as mean ± SD (*n* = 3; *p ≤ .05).

We evaluated mesothelin expression in tumour biopsies of several cancer types including GI cancers (Table S1) using the publicly available GEPIA (http://gepia.cancer‐pku.cn/) using TCGA database for tumour and normal matched tissue. When compared to normal tissues, mesothelin expression was increased in most tumours (Figure S1A). We specifically compared gastric and colon tumours with normal matched tissues and found that mesothelin expression was significantly higher in both these cancer types with respect to expression in normal tissues (*p* < .005) as shown in the boxplots in Figure 1A. When we repeated the analysis by using both TCGA and normal tissues samples from GTEX data base, we got similar results (Figure S1B). To explore the association between mesothelin expression and prognosis of patients with gastric and colon cancers, we generated Kaplan–Meyer survival curves of patients with high and low MSLN expression based on the TCGA database for survival analysis. Our results indicated that the expression levels of mesothelin was closely associated with the survival rate of patients with gastric cancer but not with colon carcinoma (Figure 1B). Low levels of mesothelin expression in gastric cancer exhibited a notable correlation with both improved overall survival (OS) (*p* = .051) (Figure 1B, left panel) and disease‐free survival (DFS) (*p* = .067) (Figure S1C). However, for colon carcinoma, there was no significant difference in OS (Figure 1B, right panel) and DFS (Figure S1D) rates between high and low mesothelin expression groups (*p* = .3 and *p* = .43, respectively). These results show that while high MSLN expression is associated with improved overall survival in gastric cancer it is associated with poor survival in colorectal cancers. At the present time, studies of other tumour types have also shown conflicting data regarding the association of MSLN expression with survival.^30^ Specifically, studies of MSLN expression in gastric cancer and overall survival have shown contradicting results^31^ that are probably a result of study methodology, and only prospective studies can fully address this question. The observed correlation between mesothelin expression and survival in gastric cancer may be used as a prognostic biomarker for this tumour.

Next, we evaluated the cell surface expression of mesothelin by flow cytometry using a panel of gastric and colorectal cancer cell lines. Gastric cancer cell lines AGS, N87, and HGC27, along with colorectal cancer cell lines SW48 and HTB39, tested positive for MSLN expression (Figure 1C). The data revealed a diverse expression pattern of mesothelin across these cell lines. Most of these cell lines displayed medium to high mesothelin expression and the number of mesothelin molecules per cell ranged from 10 000 to 71 000 (Figure 1D). Notably, N87 and SW48 had the highest MSLN expression among the gastric and colorectal cancer cell lines assessed, with average number of molecules per cell of 71 000. Additionally, all the gastric and colorectal cancer cell lines used in the study express PDL1 (Figure S1E and F). Taken together, these studies demonstrate that mesothelin is highly expressed in gastric and colon tumours as well as in cell lines, making them attractive candidates for mesothelin‐targeted CAR‐T cell therapies.

### 3.2 Gastric and colorectal cancer cell lines expressing mesothelin are sensitive to killing by hYP218 CAR T cells

We generated hYP218 CAR T cells from three healthy donor PBMCs (donor 1, donor 2 and donor 3) by lentiviral transduction of hYP218 CAR construct (Figure S2A) and determined the transduction efficiency, in vitro CAR‐T cell expansion, and CD4+/CD8+ ratio of the CAR T cells. Transduction efficiency was 74.5% for donor 1, 52.9% for donor 2 and 27.4% for donor 3 (Figure S2B). The fold expansion on day 9 for hYP218 CAR T cells was 73‐fold, 96‐fold, and 107‐fold for donors 1, 2, and 3, respectively (Figure S2C). Our results revealed efficient transduction and expansion of T cells. In addition, the CD4/CD8 ratio increased for donors 1 and 2 compared to untransduced T cells. However, the CD4+/CD8+ ratio was comparable between untransduced and hYP218 CAR‐transduced T cells for donor 3 (Figure S2D and E).

**FIGURE 2:**
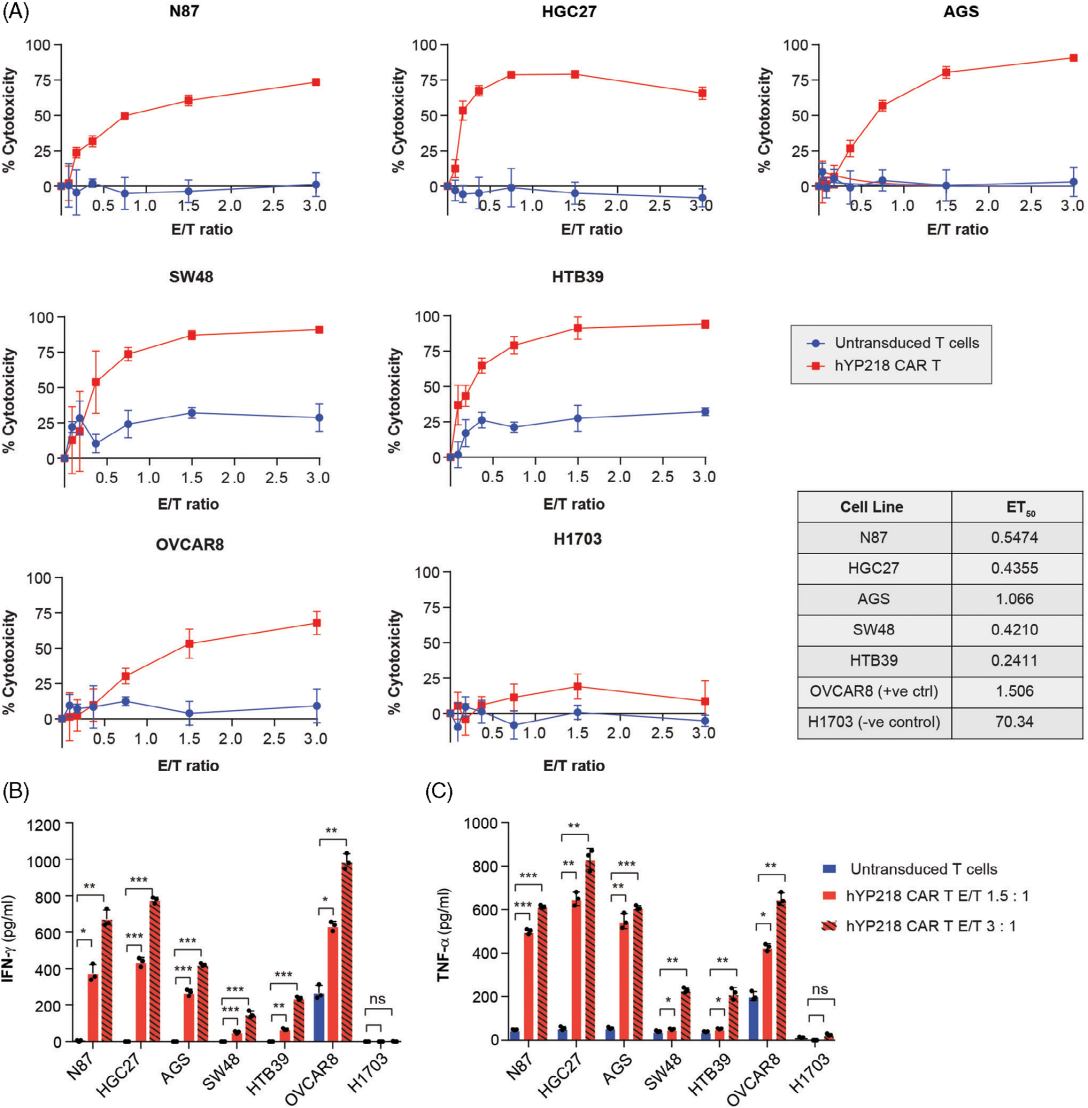
Cytotoxicity of hYP218 CAR T cells against gastric and colorectal cancer cell lines. (A) Cytotoxicity of hYP218 CAR T or untransduced T cells upon co‐culture at different E:T ratios against mesothelin expressing gastric (N87, HGC27 and AGS) and colorectal tumour cell lines (SW48 and HTB39). OVCAR‐8 and H1703 cells were used as positive and negative control, respectively for mesothelin expression. ET_50_ values for each cell line shown in the table. (B) Release of IFN‐γ and (C) TNF‐α after co‐culture of hYP218 CAR T or untransduced T cells with tumour cells at E:T ratio of 1.5:1 and 3:1. Data represent ± SD (*n* = 3). Significance levels: ns = non‐significant; *p ≤ .05; **p ≤ .01; ***p ≤ .001.

Cytotoxicity of hYP218 CAR T cells, generated from donor 1, was assessed by in vitro coculture assay to measure cell killing at different effector to target (E:T) ratios and by cytokine release (Figure 2). All the gastric and colorectal cancer cell lines tested showed cell killing at low E:T ratios similar to the mesothelin expressing OVCAR8 cells used as a positive control (Figure 2A). The ET50 for OVCAR8 was 1.5 whereas that for the other cell lines varied from 0.24 (HTB39) to 1.0 (AGS). H1703, a MSLN‐negative control cell line, was not sensitive to killing by YP218 CAR T cells with ET50 of 70.34. This indicates a target‐specific lysis of cancer cells by hYP218 CAR T cells. There was no correlation between the extent of CAR T cell‐mediated cytotoxicity and the expression levels of MSLN molecules on the tested cell lines. This indicates that hYP218 CAR T cells can effectively kill target cells, as demonstrated by the similar sensitivity of the AGS cell line (10 822 MSLN molecules/cell) and SW48 cell line (71 092 molecules/cell), while H1703 cell line, which lacks MSLN expression, was resistant. Cytokine release evaluated at E:T ratios of 1.5 and 3 revealed a significant increase in the production of pro‐inflammatory cytokines, INF‐γ and TNF‐α by the CAR T cells compared to untransduced T cells (Figure 2B and C). The magnitude of cytokine release was higher at increased E:T ratio, indicating a correlation between effector cell density and cytokine secretion. Similar results regarding cytotoxicity and cytokine release were obtained using hYP218 CAR T cells generated from donors 2 and 3 (Figure S3A–F). The observation that some cell lines with lower mesothelin expression induced higher cytokine secretion from CAR‐T cells may be attributed to intrinsic differences in the cell lines, including variations in co‐stimulatory molecule expression.^32^ We next evaluated the activation and exhaustion phenotypes of hYP218 CAR T cells 24 h post‐coculture with tumour cells. hYP218 CAR T cells were enriched for CD69 and CD39 activation markers, with 22.5% and 14.8%, respectively, as compared to 12.8% and 4.2% in the untransduced T cells. In addition, a slight increase in the expression of exhaustion markers PD1, TIM3, and lymphocyte‐activation gene 3 (LAG3) was observed. Human CD3+ cells, gated on live cells, were used for CAR T cell analysis, assessing activation and exhaustion markers (Figure S4A–C). Taken together, our results demonstrate that CAR T cells are effectively activated upon encountering MSLN+ target cells, with minimal signs of exhaustion and increased activation.

### 3.3 hYP218 CAR T cells remain functional despite repetitive antigen exposure

**FIGURE 3:**
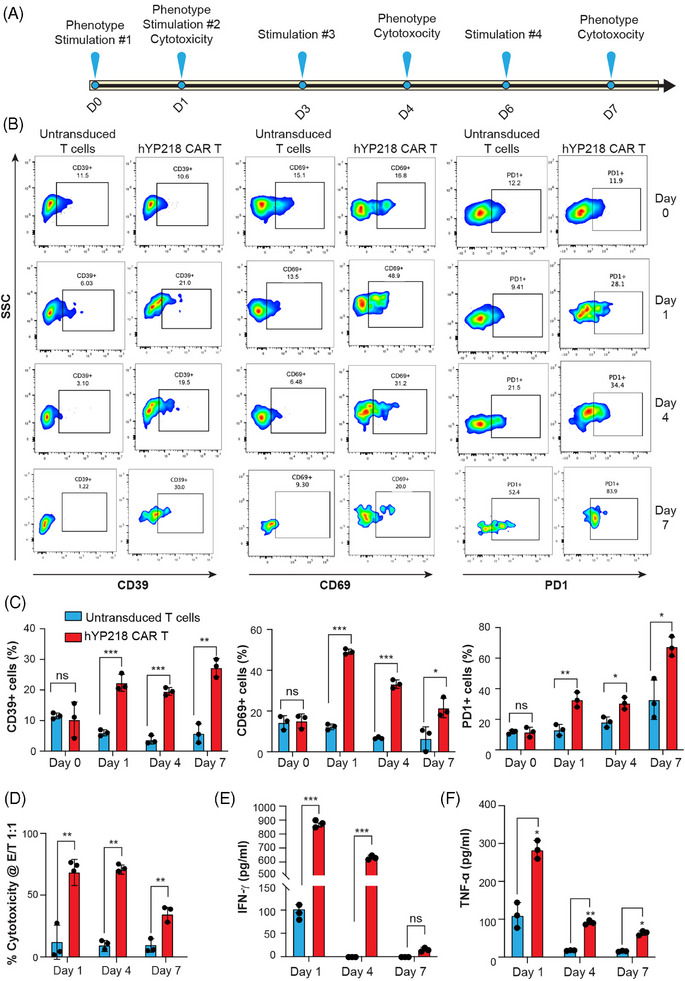
Phenotypic changes and cytotoxicity of hYP218 CAR T cells after in vitro antigen restimulation. (A) Schematic diagram of tumour cells and T cells co‐culture assay. (B, C) Activation (CD39 and CD69) and exhaustion (PD1) markers expressed by hYP218 CAR T cell products as determined by flowcytometry. The status of activation and exhaustion markers were assessed on day 0 and following antigen restimulation using HGC27 target cells on days 1, 4, and 7. (D) The percentage of HGC27 MSLN+ target cells killed by hYP218 CAR T cells was assessed after a single exposure on day 1, at a mid‐time point on day 4, and following four cycles of antigen exposure on day 7. (E, F) ELISA quantification of (E) IFN‐γ and (F) TNF‐α release by hYP218 CAR T cells was performed upon coculture with target cells on days 1, 4, and 7. Gating strategy: FSC/SSC > Singlets > Live > CD3+ > EGFR+ > CD39/CD69+/PD1+. Data are plotted as mean ± SD (*n* = 3). Significance levels: ns = non‐significant; *p ≤ .05; **p ≤ .01; ***p ≤ .001.

Having shown that hYP218 CAR T cells are activated after brief antigen encounter (Figure S4A–C) we evaluated their phenotype and functional activity after repeated antigen stimulation, similar to what would be seen in patients. To do so, hYP218 CAR T cells were repeatedly cocultured with HGC27 cancer cells at E:T ratio of 1:1 on days 0, 1, 3 and 6 as shown in Figure 3A. The phenotype and killing ability of hYP218 CAR T cells was determined on day 0 (before coculture with tumour cells, for determination of phenotype), day 1, day 4, and day 7. At day 7 post coculture, we observed that the hYP218 CAR T remained activated as indicated by the sustained levels of activation markers CD39+ (10.6% on day 0 to 30% on day 7) and CD69+ (16.8% on day 0 to 20.0% on day 7). Of note, the values for CD69 were higher on day 4. However, there was a significant increase in PD1 expression, from 11.9% on day 0 to approximately 80% on day 7 (Figure 3B and C). Similarly, a significant increase in the expression levels of TIM3 and LAG3 was observed (Figure S5A–C). More importantly, the hYP218 CAR T cells maintained their cytotoxicity against MSLN+ HGC27 cancer cells even at day 7 (Figure 3D). There was increased release of the effector cytokines IFN‐gamma and TNF‐alpha compared to untransduced T cells at all time points. There was a decrease in the levels on day 7 especially for IFN‐gamma (Figure 3E and F). Similar results were obtained when hYP218 CAR T cells were repeatedly stimulated with SW48 cancer cells (Figure S5D–L). Moreover, hYP218 CAR T cells expanded and proliferated more than untransduced T cells, as demonstrated by the fold expansion and the percentage of CFSE dim cells (Figure S6A–C). The fold expansion for hYP218 CAR T cells was 2.5 and 1.5 on days 1 and 7, respectively, whereas for the untransduced T cells, it was 0.4 and 0.06. These results were further supported by the CFSE assay, which showed greater cell division in hYP218 CAR T cells (93.5% CFSE dim cells) compared to untransduced T cells (58.6% CFSE dim cells). Additionally, the hYP218 CAR T cells showed a gradual decrease in the expression of memory‐like CD45RA+CD62L+ markers from day 0 to day 1 (Figure S6D and E). These findings suggest that hYP218 CAR T cells maintained their cell killing ability despite increased PD1 expression on repetitive antigen stimulation.

### 3.4 hYP218 CAR T cells lead to tumour regression and improved survival in mouse models of gastric and colorectal carcinoma

**FIGURE 4:**
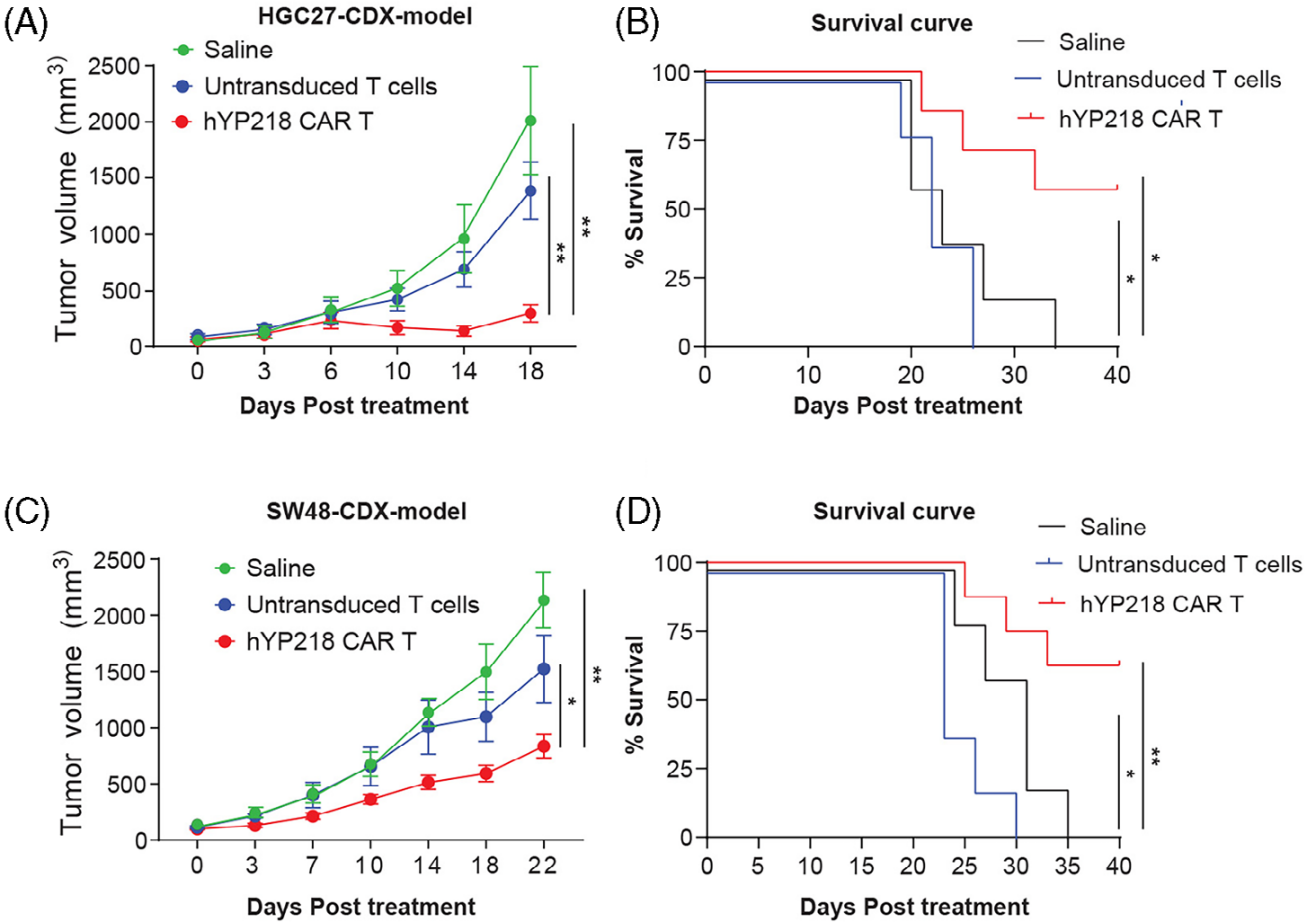
In vivo anti‐tumour efficacy of hYP218 CAR T cells using mesothelin expressing gastric and colorectal tumour models. (A) Kinetics of HGC27 gastric tumour growth in saline, Untransduced T cells and hYP218 CAR T cell treated mice. NSG mice were injected with 1 × 10^6^ cells. After 14 days, they were treated either with 1 × 10^6^ of hYP218 CAR T cells, or mock T cells or saline (*n* = 5 per treatment group). Tumour growth was monitored, and tumour volume was measured twice a week. (B) K–M survival curve shows survival benefit of the hYP218 CAR T treated mice (*p* ≤ .05 hYP218 CAR T cell versus Untransduced T cells, median survival > 40 days). (C) Kinetics of SW48 colorectal cancer tumour growth in NSG mice. Mice were injected with 1 × 10^6^ cells SW48 tumour cells and 12 days later were treated with 1 × 10^6^ of hYP218 CAR T cells, or mock T cells or saline (*n* = 5 per treatment group). (D) K–M survival curve shows survival benefit of the hYP218 CAR T treated mice (*p* ≤ .001 hYP218 CAR T cell versus Untransduced T cells, median survival > 40 days). Data represent mean ± SEM. Significance levels: ns = non‐significant; *p ≤ .05; **p ≤ .01; ***p ≤ .001.

The in vivo efficacy of hYP218 CAR T cells was evaluated in two different cancer xenograft models, HGC27 and SW48. NSG mice were injected with 1 × 10^6^ cancer cells and the tumours were allowed to grow until they reached a volume of 80–100 mm^3^, after which the mice were treated with either 5×10^6^ hYP218 CAR T cells, 5×10^6^ untransduced T cells, or saline and monitored over time. Five to seven mice were included in each group. In the HGC27‐tumour model, hYP218 CAR T cell treatment resulted in a significant reduction in tumour volume compared to both saline and untransduced T cell treated mice (Figure 4A). In addition, there was significantly improved survival of mice treated with hYP218 CAR T cells with a median overall survival greater than 40 days compared to 21 days in saline and untransduced T cell treated mice (Figure 4B). Similarly, in the SW48 tumour model, treatment with hYP218 CAR T cells led to greater tumour regression and improved survival compared to mice treated with saline or untransduced T cells (Figure 4C and D). The tumour volumes of each mouse were measured until they reached the humane endpoint, with the saline and untransduced T cell groups reaching this point early and being sacrificed accordingly (Figure S7A and B). The mice were monitored for body weight changes, with no significant changes noted throughout the observation period (Figure S7C and D). Taken together, hYP218 CAR T cells demonstrated antitumour efficacy with improved overall survival in aggressive HGC27 and SW48 tumour xenografts where the saline treated mice reached a tumour size of about 2000 mm^3^ by day 18 to 22 and had to be euthanised as per institutional ACUC guidelines. The HGC27‐tumour model showed greater tumour control, with complete tumour regression in 2 out of 7 mice (28%) treated with hYP218 CAR T cells whereas, in the SW48‐tumour model, hYP218 CAR T cells did not result in complete tumour regression.

### 3.5 Long‐term persistence of activated hYP218 CAR T cells in tumours and spleen of treated mice

**FIGURE 5:**
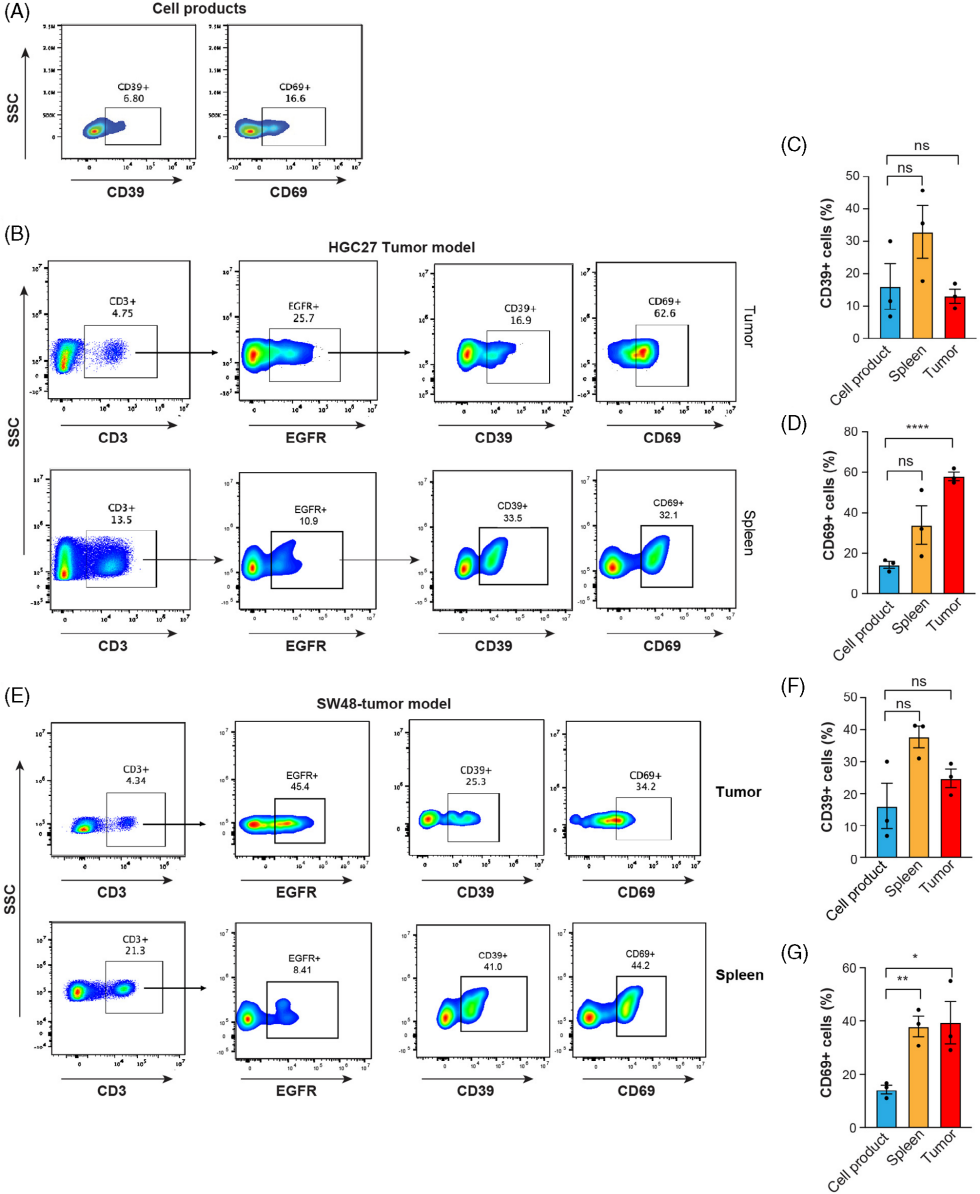
Long‐term persistence of activated hYP218 CAR T cells in tumour and spleen of treated mice. (A) Flow cytometry analysis showing the basal expression levels of activation markers CD39 and CD69 in CAR T cell products on day 9 posttransduction. (B–G) Flow cytometry analysis showing the presence of CD3+ EGFR+ cells along with their activation phenotypes in the tumour and spleen collected on day 40 after CAR T cell infusion in mice bearing (B–D) HGC27 and (E–G) SW48 tumours. Gating strategy:FSC/SSC > Singlets > Live > CD3+ > EGFR+ > CD39/CD69+. Data are plotted as mean ± SD, ns = non‐significant; *p ≤ .05; **p ≤ .01; ****p ≤ .0001.

**FIGURE 6:**
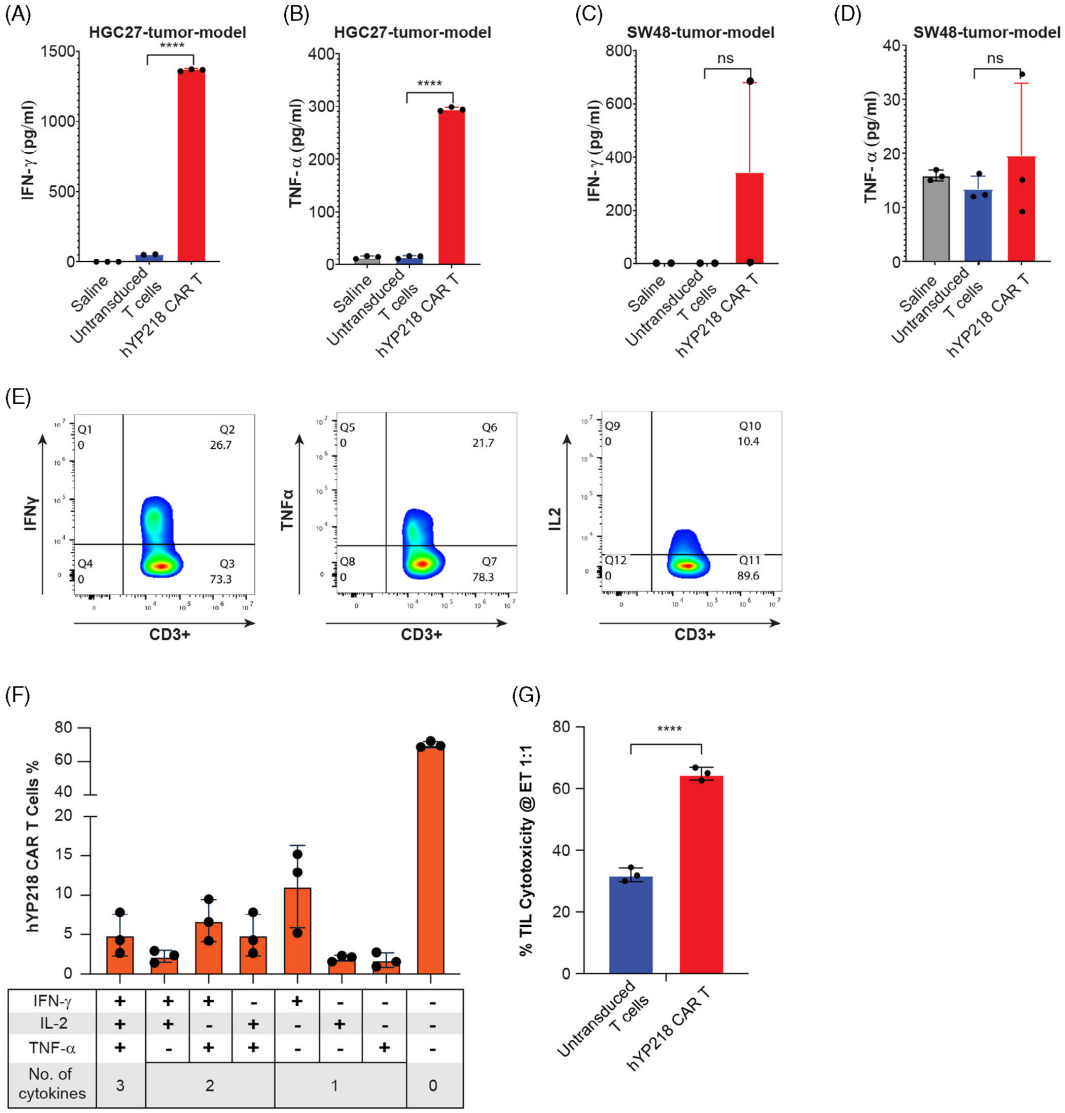
Functional status of the tumour infiltrating hYP218 CAR T cells. (A–D) TNF‐α and Interferon‐γ levels in the serum of hYP218 CAR T treated mice bearing HGC27 and SW48 tumours, collected on day 40 post treatment. A significantly higher concentration of both cytokines was observed in the HGC27 compared to the SW48 tumour models. Data are plotted as mean ± SEM (*n* = 3; *p* ≤ .0001 hYP218 CAR T versus Untransduced T cell in HGC27 cancer model; *p* ≥ .05 hYP218 CAR T versus Untransduced T cell in SW48 cancer model). (E, F) Effector cytokines released by hYP218 CAR T cells upon stimulation with Leukocyte Activation Cocktail for 4 h. (G) Cytotoxicity caused by the isolated tumour‐infiltrated hYP218 CAR T cells and untransduced T cells over a 24‐h time period at a 1:1 effector‐to‐target ratio. Gating strategy: FSC/SSC > Singlets > Live > CD3+ > EGFR+ > IFN+/TNF+/IL2+. Data are represented as mean ± SD, ns = non‐significant; ****p ≤ .0001.

Since the lack of persistence of CAR T cells is a hindrance to treating solid tumour, we evaluated the presence of activated hYP218 CAR T cells in tumour, blood and spleen at day 40, post treatment in HGC27 and SW48 NSG mouse tumour models. To do so, we evaluated the expression of activation markers CD39+ and CD69+ on the CAR‐T cell product (Figure 5A) as well as on CAR T cells isolated from tumour and spleen of treated mice at day 40, post treatment (Figure 5B–G). The distribution of CAR T cells was substantially different between tumour and spleen in HCG‐tumour model. The tumour contained 25.7% (± 1.84) CAR T cells (gated on CD3+ cells), indicating successful targeting and infiltration of these cells to the tumour site. In contrast, the spleen contained only 10.9% (± 2.82) CAR T cells. CD39 expression on the surface of hYP218 CAR T cells was 16.9% (± 3.10) in the tumour tissue and 33.5% (± 11.54) in the spleen. Similarly, CD69 was expressed on 62.6% (± 2.94) and 32.1% (± 13.52) of hYP218 CAR T cells isolated from the tumour and spleen tissue, respectively. For the SW48‐tumour model, the percentage of hYP218 CAR T cells in the tumour tissue and spleen was 45.4% (± 5.01) and 8.41% (± 4.4), respectively. This showed a similar trend to the HGC27‐tumour model, with CAR T cells preferentially localising to the tumour site. CD39 was expressed on 25.3% (± 4.06) of hYP218 CAR T cells isolated from tumour tissue and 41.0% (± 4.76) from the spleen. The higher expression of CD39 on CAR T cells in the spleen compared to those in the tumour likely reflects the more active immune environment of the spleen, while the tumour microenvironment's inhibitory signals, such as TGF‐β, hypoxia, and regulatory cells, may suppress CAR T cell activation and result in lower CD39 expression.^33^, ^34^ Additionally, CD69 was expressed on 34.2% (± 11.26) of hYP218 CAR T cells in tumour tissue and 44.2% (± 5.52) in the spleen. This indicates that the hYP218 CAR T cells isolated from both the xenograft models displayed higher expression levels of activation markers CD39 and CD69 when compared to the cell product used for treatment indicating an ongoing state of activation. Although hYP218 CAR T cells were detected in both in the spleen and tumour at day 40 after injection, the per cent of CAR T cells was more in the tumour than in the spleen. This shows tumour infiltration, expansion, and persistence of hYP218 CAR T cells. Furthermore, we attempted to analyse CAR T cell presence in the blood, but the cell numbers were either undetectable or too low for analysis. Nevertheless, we analysed the blood serum for effector cytokines and successfully detected the presence of IFN‐γ and TNF‐α. A concomitant elevation in the levels of effector cytokines, including interferon‐gamma (IFN‐γ) and tumour necrosis factor‐alpha (TNF‐α) was detected in the blood of treated mice in both models (Figure 6A–D). These cytokine levels were significantly elevated compared to mice that were treated with saline or untransduced T cells demonstrating the functional capacity of the CAR T cells to not only persist but also to maintain an activated state. In addition, we conducted further experiments to assess whether the tumour‐infiltrated hYP218 CAR T cells were functional. Tumour‐infiltrating lymphocytes (TILs) were isolated, and their functional capacity was evaluated using a leukocyte activation cocktail with BD GolgiPlug (BD Biosciences). Upon stimulation with the leukocyte activation cocktail, the isolated hYP218 CAR T cells produced INF‐γ, TNF‐α and IL2. Boolean gate analysis revealed that 5% of the hYP218 CAR T cells could produce all three effector cytokines, while the percentages of CAR T cells secreting only INF‐γ, TNF‐α and IL2 were 11.1%, 2%, and 1.74%, respectively (Figure 6E and F). This approach allowed us to measure effector cytokine production and activation status, providing insight into the functional activation of the infiltrating cells. Human CD3+ cells, gated on live cells, were used to analyse hYP218 CAR T cells and effector cytokines (Figure S8). Moreover, we performed direct co‐culture assays using HGC‐27 target cells to assess the cytotoxic activity of the isolated TILs. The isolated hYP218 CAR T cells induced a cytotoxicity of 64.7% (± 1.72), compared to 32% (± 1.85) induced by the untransduced T cells (Figure 6G). These functional assays, combined with the phenotypic data, provide a comprehensive understanding of the functional state of the infiltrated hYP218 CAR T cells.

### 3.6 Upregulation of PD1 and TGFBR2 on tumour infiltrating and spleen resident hYP218 CAR‐T cells

**FIGURE 7:**
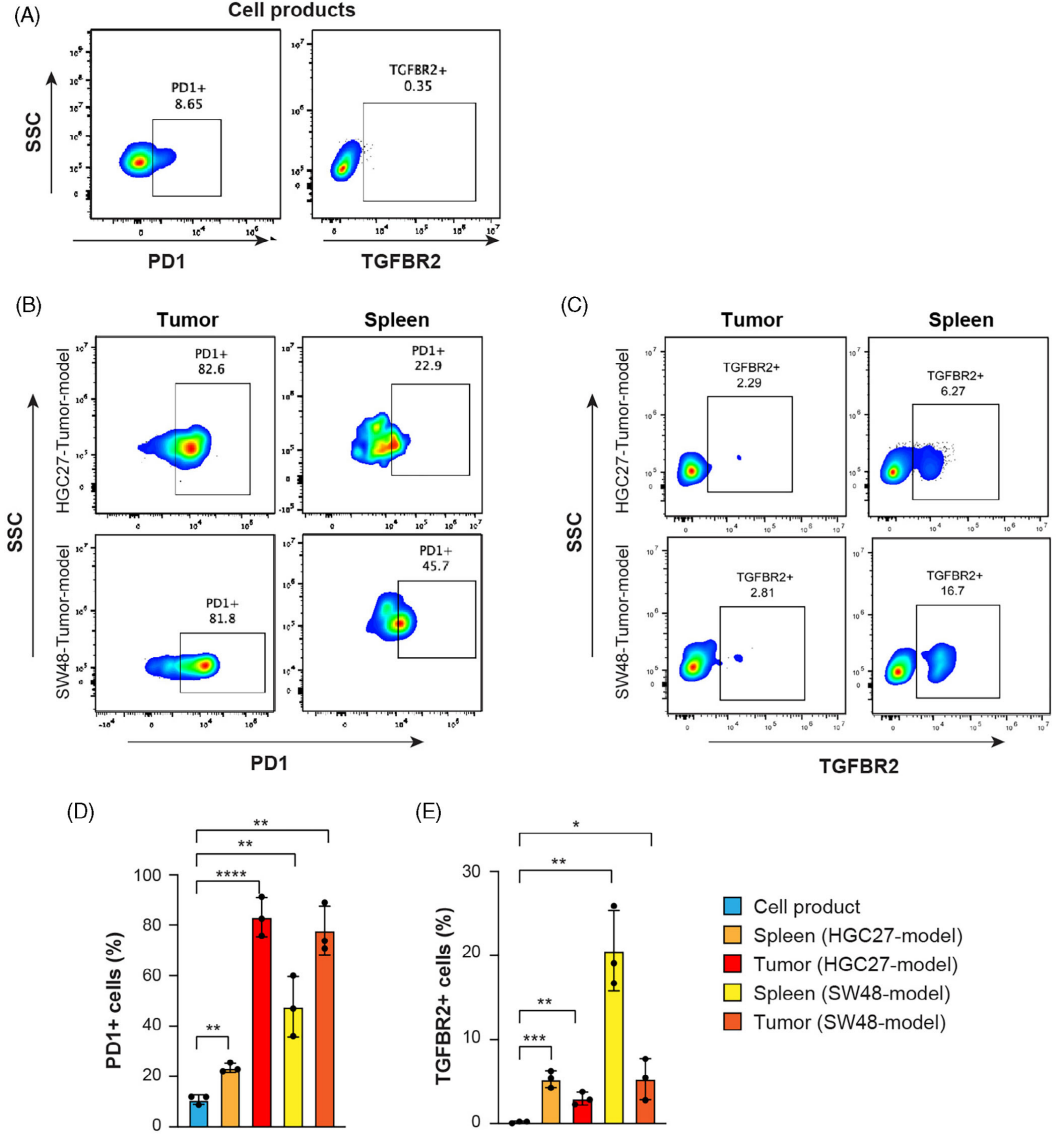
Expression of exhaustion markers on hYP218 CAR T cell product and CAR T cells isolated from tumour and spleen of treated mice. (A) Representative flow cytometry data showing basal expression of PD1 and TGFBR2 in hYP218 CAR T cell products. Low expression of PD1 was seen across the samples, while TGFBR2 expression was undetectable. (B) Representative flow cytometry dot plots depicting the elevated expression of PD1 in CAR T cells isolated from tumour and spleen tissues of HGC27 and SW48 tumour bearing mice on day 40 post hYP218 CAR T treatment when compared with the baseline expression seen in the cell product (*n* = 3). (C) Flow Cytometry analysis showing increased TGFBR2 expression on CAR T cells isolated from the tumour and spleen tissues of mice bearing HGC27 and SW48 tumours that were treated with hYP218 CAR T cells (*n* = 3). (D, E) Cumulative bar plots showing the percentage of PD1 and TGFBR2‐positive cells in cell products, spleen, and tumour of HGC27 and SW48 CDX models, respectively. Gating strategy: FSC/SSC > Singlets > Live > CD3+ > EGFR+ > PD1/TGFBR2+. Data are plotted as mean ± SD, *p ≤ .05; **p ≤ .01; ***p ≤ .001.

While we observed tumour regression in most of the mice treated with hYP218 CAR T cells as well as improved overall survival, complete tumour regression was rare. This lack of complete tumour regression prompted us to investigate the underlying reasons and potential mechanisms. Similar to the result of our in vitro repeated co‐culture assay, PD1 expression levels on hYP218 CAR T cells in both spleen and tumour were remarkably increased with approximately 80% of hYP218 CAR T cells isolated from the tumour expressing PD1 on day 40 compared to 8.6% on the hYP218 CAR T cells that were injected to the mice (Figure 7A, B and D). Additionally, TGFBR2 expression on the hYP218 CAR T cells which was undetectable in the cell products, increased to significantly detectable levels posttreatment (Figure 7A, C and E). In contrast, the expression levels of other coinhibitory molecules such TIM‐3 and LAG‐3 remained relatively stable, showing no significant changes over the same period. Human CD3+ cells, gated on live cells, were used for analysing hYP218 CAR T cells and inhibitory markers (Figure S9A–F). These findings suggest that while the CAR T cells remain functionally active, they are also experiencing a form of adaptive resistance marked by the overexpression of specific inhibitory receptors.^35^

### 3.7 Combining hYP218 CAR T cells with the anti‐PD1 antibody pembrolizumab enhances anti‐tumour efficacy

**FIGURE 8:**
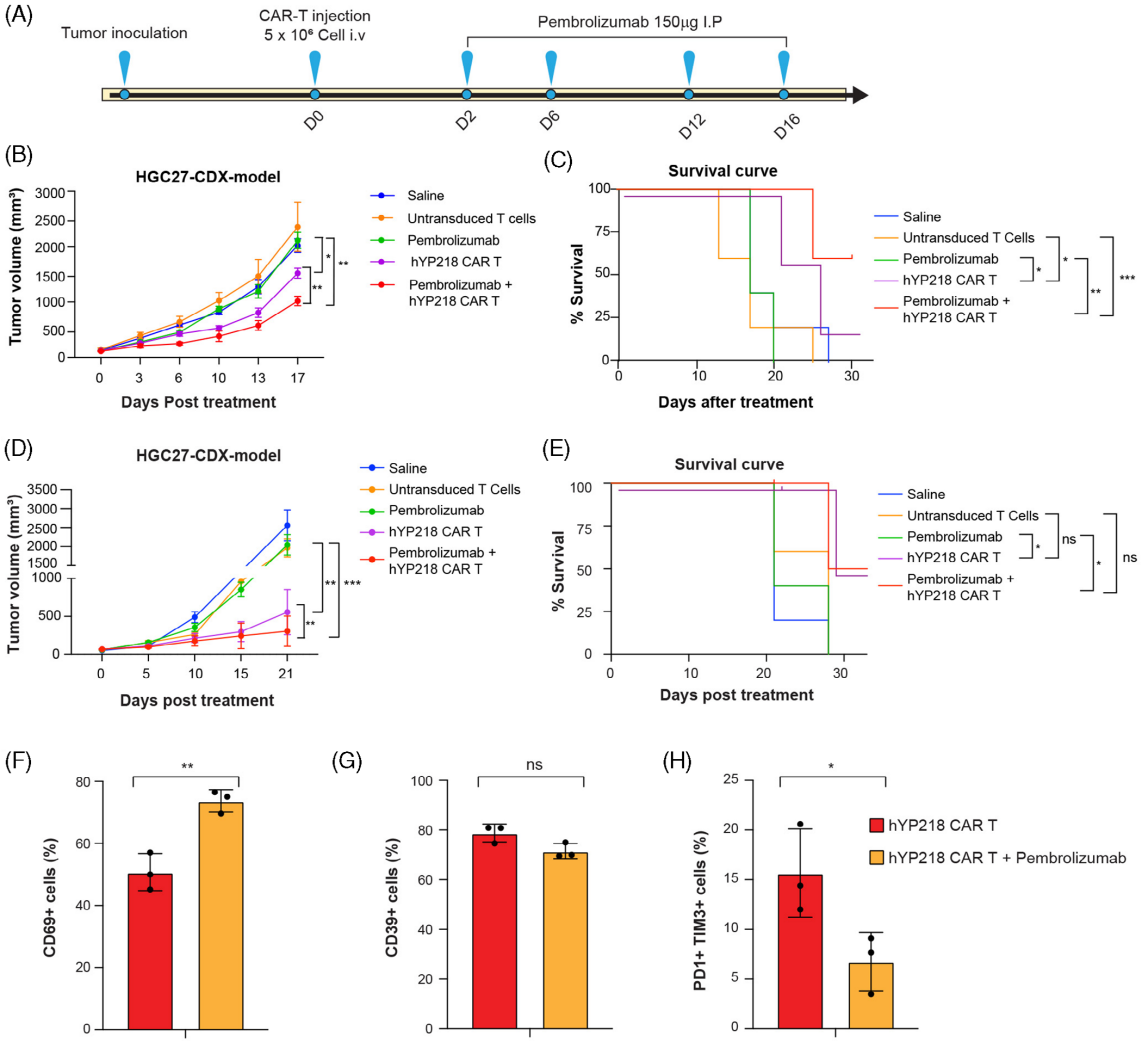
The combination of hYP218 CAR T cells with the anti‐PD1 antibody pembrolizumab enhances tumour control. (A) The experimental design of the in vivo experiment involving hYP218 and the anti‐PD1 antibody was as follows: 1 × 10^6^ HGC27 cancer cells were injected per mouse, and treatment began when the tumour volume reached 150 mm^3^. The treatment consisted of a single dose of 5 × 10^6^ hYP218 CAR T cells followed by four doses of the anti‐PD1 antibody (pembrolizumab). (B) Kinetics of HGC27 gastric tumour growth in saline, mock, pembrolizumab and hYP218 CAR T cell treated mice. NSG mice were injected with 1 × 10⁶ cells and allowed to grow until more robust, larger tumours of approximately 150 mm^3^ had formed. Mice were treated either with 5 × 10^6^ of hYP218 CAR T cells, pembrolizumab (150µg) or mock T cells or saline (*p* ≤ .001 combination group versus hYP218 CAR T cell, *n* = 5 per treatment group). Tumour growth was monitored, and tumour volume was measured twice a week. (C) Survival curve shows survival benefit of the combined hYP218 CAR T and pembrolizumab treated mice (*p* ≤ .05 pembrolizumab group versus hYP218 CAR T cell, *p* ≤ .001 pembrolizumab group versus combination group, *n* = 5 per treatment group). (D) Kinetics of HGC27 gastric tumour growth in saline, Untransduced T cells, pembrolizumab, hYP218 CAR T, and the combination group. NSG mice (*n* = 5 per treatment group) were injected with 1×10⁶ cells, and treatment was initiated when the tumour volume was small, approximately 80 mm^3^. Tumour growth was monitored, and tumour volume was measured twice a week. (E) Kaplan–Meier survival curve. (F–H) Flowcytometry data showing the expression of CD69, CD39 and PD1‐TIM3 expression on hYP218 CAR T cells in the pembrolizumab‐treated group compared to the group that did not receive pembrolizumab. Data are represented as mean ± SD, ns = non‐significant; *p ≤ .05; **p ≤ .01; ***p ≤ .001.

Since PD1 expression was significantly increased on tumour infiltrating hYP218 CAR T cells, we next investigated whether treatment with the anti‐PD1 antibody pembrolizumab would increase the efficacy of hYP218 CAR T cells. HGC27 cancer cells were inoculated into NSG mice and to better determine the benefit of adding pembrolizumab to CAR T cell treatment we treated larger and more established tumours of around 150 mm^3^. A single dose of 5×10^6^ million hYP218 CAR T cells was administered, followed by four doses of 150 µg of pembrolizumab on days 2, 6, 12, and 16 (Figure 8A). The combination of hYP218 CAR T cells with pembrolizumab demonstrated slower tumour growth compared to mice treated with hYP218 CAR T cells alone (Figure 8B). Furthermore, the combination of pembrolizumab with hYP218 significantly extended the survival of the mice when compared to mice treated with hYP218 CAR T cells pembrolizumab alone. The median overall survival increased from 25 days in the hYP218 CAR T cell group to over 50 days in the combination group (Figure 8C). However, there we did not see any complete responses.

Furthermore, we performed an additional combination experiment, where treatment was initiated when tumours were smaller, tumour volume of approximately 80 mm^3^, to determine the impact of combining CAR T cells with pembrolizumab. As shown in Figure 8D and E, the potent tumour control exerted by hYP218 CAR T cells alone in these smaller tumours obscured any discernible advantage of the combination therapy with pembrolizumab. Tumour volumes for each mouse were monitored until they reached the humane endpoint, with the saline, pembrolizumab only and untransduced T cell groups reaching this threshold early and being sacrificed (Figure S10A and B). Additionally, our findings revealed that hYP218 CAR T cells in pembrolizumab‐treated mice increased the expression of the activation marker CD69 and lowered the expression of PD1/TIM3 double‐positive exhaustion signature compared to the group receiving hYP218 CAR T cells alone (Figure 8F and H). The expression of CD39 was similar between the two groups (Figure 8G), whereas the expression of PD‐1, TIM3, and LAG3 was lower in the combination group, although not statistically significant (Figure S10C–E). These findings highlight the potential synergy between CAR T cells and pembrolizumab in enhancing tumour control in larger established tumours but not smaller tumours which are very sensitive to CAR T cell treatment alone.

## 4 DISCUSSION

Although there have been advances in the treatment of patients with gastric and colorectal cancers, the prognosis of patients with metastatic disease after progression on standard therapies is dismal.^6^, ^36^ Therefore, there is a need to develop novel and efficacious treatments for these patients. In our study, we have characterised the expression of the tumour differentiation antigen mesothelin in these cancer types and demonstrated the efficacy of the mesothelin‐targeted hYP218 CAR T cells in gastric and colorectal cell lines and tumour xenografts. More importantly, we have characterised the phenotype of CAR T cells persisting in the tumours and showed that they developed adaptive resistance mechanism by over expressing PD1, which can be partly overcome with anti‐PD1 checkpoint inhibitor treatment.

Our prior studies have shown that hYP218 CAR T cells, targeting the cell membrane‐proximal epitope of mesothelin, demonstrate significant anti‐tumour efficacy in preclinical models of mesothelioma, ovarian and pancreatic cancers. Given the superior efficacy and persistence of hYP218 CAR T cells compared to SS1 CAR T cells, which target the membrane‐distal region of MSLN, we believe that hYP218 CAR T cells may offer enhanced therapeutic potential for patients.^28^ Previous clinical studies using anti‐mesothelin CAR T cells that used the SS1 scFv showed limited effectiveness in patients.^20^, ^37^, ^38^ To expand the potential utility of hYP218 CAR T cells in the clinic for the treatment of common solid tumours, we used the public database GEPIA to evaluate mesothelin expression in GI cancers. Our results show high expression of mesothelin mRNA in biopsies of patients with gastric and colon cancers, as compared with matched normal tissues. This is in line with prior studies using IHC that have shown mesothelin expression in approximately 47% of gastric cancer cases and about 30% of colorectal cancers.^30^, ^39^, ^40^, ^41^ Our results also show that lower mesothelin expression is associated with increased overall survival in gastric carcinomas but not colon carcinoma. Our results are similar to previous studies that show variable effects of mesothelin expression on patient outcomes in gastric and colon cancers.^25^, ^26^, ^27^, ^42^

We also confirmed mesothelin expression in a variety of established gastric and colorectal cancer cell lines and demonstrated their sensitivity to killing by hYP218 CAR T cells. The in vivo efficacy of hYP218 CAR T cells was evaluated in highly aggressive gastric and colorectal tumour models. In the gastric cancer HGC27 model, hYP218 CAR T cell treatment led to significant tumour regression, including complete tumour eradication in 28% of treated mice, and improved survival compared to mice treated with untransduced T cells. Similar results were seen in the SW48 colorectal carcinoma model, although none of the hYP218 CAR T cell treated mice had complete tumour regression. Although we have seen complete tumour eradication with hYP218 CAR T cells previously using mesothelioma, ovarian and pancreatic tumour models,^28^ in this study we mostly observed sustained tumour regression. It is possible that the lack of complete tumour response could be due to the aggressive growth rate of these tumours or the phenotypic changes of hYP218 CAR T cells in the tumour microenvironment of these tumours.

Previous studies have demonstrated the ability of anti‐mesothelin adoptive cell therapies using CAR T^43^, ^44^, ^45^, ^46^ and CAR NK cells,^47^ to specifically target and kill MSLN‐positive gastric and colorectal tumour cells. However, these studies did not explore the resistance mechanisms that may limit their therapeutic efficacy. We therefore studied the fate of CAR T cells in the tumour to tease out the mechanism of resistance. Our results show that the CAR T cells infiltrated the tumour, and we were able to detect them even 40 days after a single injection. The fact that these cells were present in higher percentages in tumours compared to the spleen, suggests that they are actively infiltrating and expanding in the tumours. In addition, the phenotypic analysis of these hYP218 CAR T cells demonstrated that they are in an activated state with significant expression of the T cell activation markers CD39 and CD69.^48^, ^49^, ^50^ However, at the same time there was increased expression of PD1 on the hYP218 CAR T cells compared to the product that was injected into mice, suggesting the development of adaptive immune resistance. However, the fact that we could detect TNF‐α and IFN‐γ in the mouse serum at day 40 after treatment suggests that these CAR T cells are functional and release cytokines in the presence of the tumour. These cytokines play critical roles in mounting effective antitumour responses. Notably, a significantly higher concentration of both cytokines was observed in the HGC27 model compared to the SW48 model. These results suggest that the hYP218 CAR T cell treatment induces a more robust inflammatory response in the HGC27 model, which might correlate with its therapeutic efficacy. The sustained activation of CAR T cells, indicated by the expression of CD39 and CD69 along with our functional assays where tumour‐infiltrating lymphocytes (TILs) were isolated and assessed for effector cytokine production and cytotoxic activity, suggests ongoing antigenic stimulation and confirms that these cells retain their anti‐tumour functionality. Whether this sustained activation enhances therapeutic efficacy in patients will be evaluated in our upcoming phase I clinical trial of hYP218 CAR‐T cells in patients with advanced solid tumours. This dynamic interplay of activated CAR T cells that also have increased PD1 expression could account for the fact that there was significant tumour regression but not complete tumour eradication. These findings are supported by studies in patients where anti‐mesothelin adoptive cell therapy using gavo‐cel led to marked PD1 expression on CAR T cells as early as seven days after infusion.^51^ Since we show that increased PD1 expression is a potential mechanism of immune resistance to hYP218 CAR T cells, we next evaluated the combination of anti‐PD1 antibody pembrolizumab plus hYP218 CAR T cells against large well established HGC27 tumours in vivo. Although there was slower tumour growth and improved overall survival with this combination treatment it did not lead to complete tumour eradication. In addition, against smaller HGC27 tumours we did not see benefit of adding pembrolizumab since hYP218 CAR T cells by themselves had significant anti‐tumour activity. Our results highlight the modest potential of combining CAR T cell therapy with anti‐PD‐1 immune checkpoint inhibition as a potential strategy for improving CAR‐T cell efficacy. These results are in line with a reported clinical study showing the disruption of the PD1 in CAR T cells mostly resulted in a stable disease rather than objective tumour regression.^52^ Similarly, addition of PD‐1 inhibition did not show any detectable change in CAR T cell expansion and patient survival in a GD2 CAR T trial in patients with neuroblastoma.^53^ Whether blocking PD1 expression by using immune checkpoint inhibitors increases the efficacy of anti‐mesothelin CAR T cells in patients, is being evaluated in a prospective clinical trial (NCT04577326).

Our study has several limitations. One of these is the use of immunodeficient mice rather than immunocompetent mice. The absence of a tumour immune microenvironment in the immunodeficient mice may alter the dynamics of the interaction between cancer cells and CAR T cells. Similarly, the onset of Graft‐versus‐Host Disease (GVHD) around day 40 of the CAR T‐cell treatment limits the duration for which the animals could be followed to better understand the impact of hYP218 CAR T cells in improving overall survival of these mice. Immunocompetent and humanised mouse models may address the limitations of immunodeficient mice and provide a more comprehensive understanding of therapy efficacy.^54^, ^55^ Although our study focused mostly on inhibiting PD‐1 to improve efficacy of hYP218 CAR T cells other mechanisms most likely play an important role in the adaptive resistances such as the release of immune suppressive cytokines like TGFβ and upregulation of TGFBR2 by CAR T cells, as was seen in our case. Inhibiting this pathway may improve the efficacy of CAR T cells in solid tumours.^35^ Furthermore, additional gastric and colorectal tumour models, each possessing different genetic and phenotypic characteristics, could be incorporated into subsequent studies to enhance our understanding of the efficacy of hYP218 CAR T cells against these cancer types. Translating findings from these models to clinical settings requires careful consideration and may not always accurately predict human responses. Therefore, as CAR T cell therapies continue to advance, it is imperative to explore strategies to mitigate exhaustion, refine animal models, and conduct comprehensive clinical trials to better understand and overcome these limitations, ultimately improving the overall success of CAR T cell‐based cancer treatments. Potential enhancements to improve CAR‐T cell efficacy for solid tumours include incorporating cytokine support (e.g., IL‐15 or IL‐12) to enhance survival, developing dual or multi‐antigen targeting to prevent tumour escape, and using CRISPR/Cas9 technology to knock out inhibitory receptors. These approaches, along with armouring CAR‐T cells with checkpoint blockade, could significantly strengthen their therapeutic potential.^56^ Lastly, although mesothelin targeted therapeutics have in general shown limited off‐tumour‐on‐target toxicity in clinical trials,^30^ it is a concern for highly active CAR T cells that can recognise low level mesothelin expression on normal tissues. Recently, two studies of highly active anti‐mesothelin CAR T cells^57^ and T cell receptor fusion^51^ construct have reported pneumonitis is some patients most likely a result of these activated T cells targeting very low mesothelin expressing cells in the lung alveoli. Therefore, future studies of anti‐mesothelin CAR T cells should carefully select patients with underlying lung disease and initiate mitigation strategies including early use of steroids to treat pneumonitis. Taken together, our study demonstrates the significant anti‐tumour efficacy of hYP218 CAR T cells in preclinical models of gastric and colorectal cancers. In vivo efficacy studies revealed notable tumour regression with hYP218 CAR T cells, though complete eradication was not mostly observed, suggesting potential resistance mechanisms, which could be further studied and targeted in combination therapies for improved outcomes.

## AUTHOR CONTRIBUTIONS

*Conceptualisation*: Sameer Mir, Abhilash Venugopalan, Qun Jiang, and Raffit Hassan. *Designing of research studies*: Sameer Mir, Abhilash Venugopalan, Qun Jiang, and Raffit Hassan. *Conducting experiments*: Sameer Mir, Abhilash Venugopalan, Jingli Zhang, Qun Jiang, and Raffit Hassan. *Acquiring data*: Sameer Mir, Abhilash Venugopalan, Jingli Zhang, Qun Jiang, and Raffit Hassan. *Analysing data*: Sameer Mir, Abhilash Venugopalan, Qun Jiang, Nishanth Ulhas Nair, and Raffit Hassan. *Writing the manuscript (original draft)*: Sameer Mir and Raffit Hassan. *Writing the manuscript (review and edit)*: Abhilash Venugopalan, Jingli Zhang, Nishanth Ulhas Nair, Manjistha Sengupta, Manakamana Khanal, Chaido Stathopoulou, Qun Jiang. *Funding acquisition*: Raffit Hassan. *Study supervision*: Raffit Hassan.

## CONFLICT OF INTEREST STATEMENT

RH has received funding from the Intramural Research Program of the NIH, National Cancer Institute, Center for Cancer Research (ZIA‐BC‐010816) and has received funding for conduct of clinical trials via a cooperative research and development agreement between NCI and Bayer AG and TCR^2^ Therapeutics. Other authors have no competing interests to disclose.

## ETHICS STATEMENT

PBMCs from healthy donors were provided by the NIH Clinical Center Department of Transfusion Medicine as part of their IRB approved and consented healthy donor program.

## CONSENT FOR PUBLICATION

All authors read and approved the final version of the manuscript.

## Supporting information



Supporting information

Supporting information

Supporting information

Supporting information

Supporting information

Supporting information

Supporting information

Supporting information

Supporting information

Supporting information

Supporting information

Supporting information

Supporting information

Supporting information

Supporting information

Supporting information

Supporting information

## Data Availability

All data relevant to the study are included in the article or uploaded as supplementary information.
