# Supplementary figures and images for: Persistence of activated anti‐mesothelin hYP218 chimeric antigen receptor T cells in the tumour is associated with efficacy in gastric and colorectal carcinomas
Source: Clin Transl Med. 2024 Nov 15;14(11):e70057. doi: 10.1002/ctm2.70057 (PMC11567854; doi:10.1002/ctm2.70057)

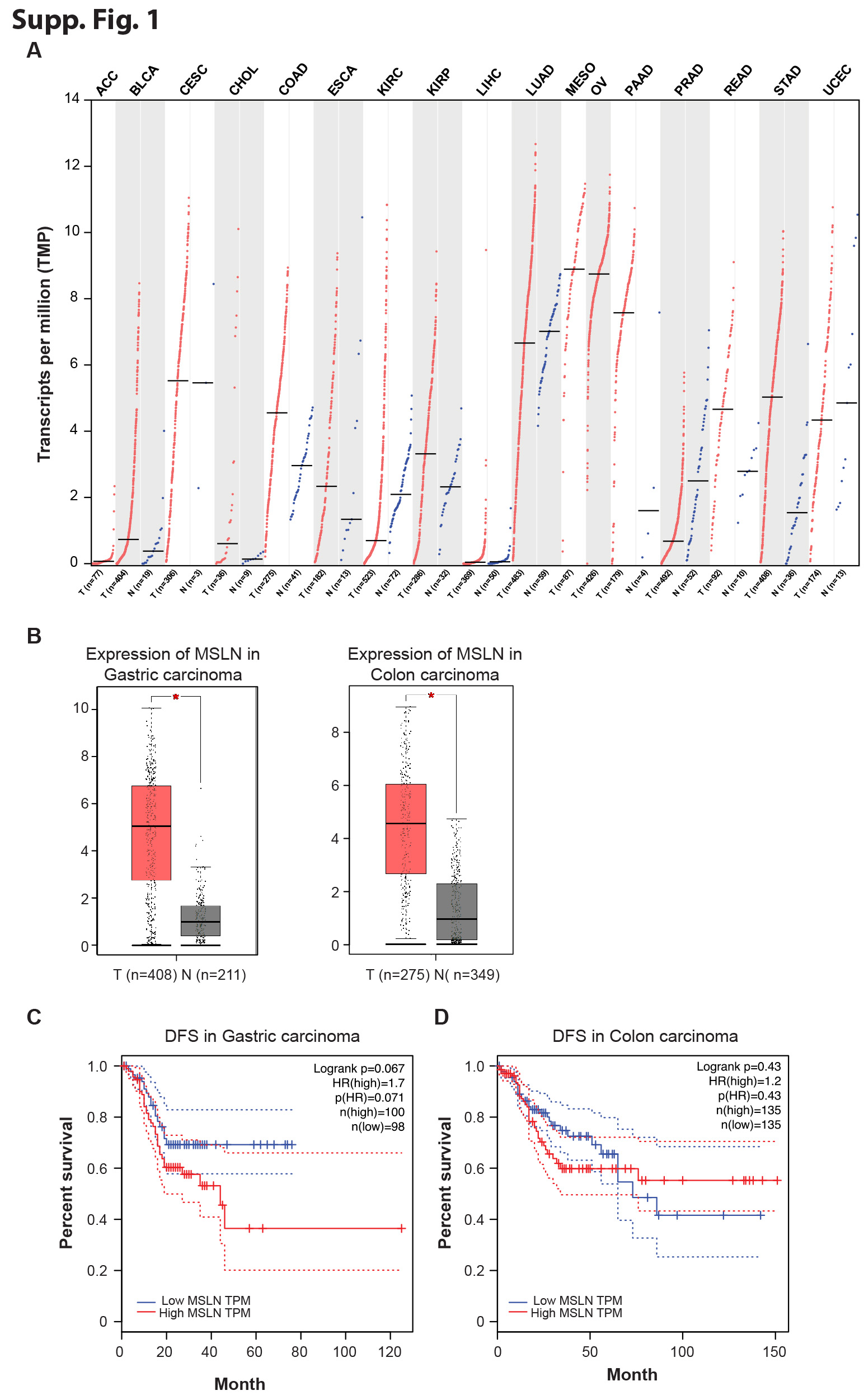

Supplement: Supplementary file 1 — Supporting information [file CTM2-14-e70057-s015.jpg]

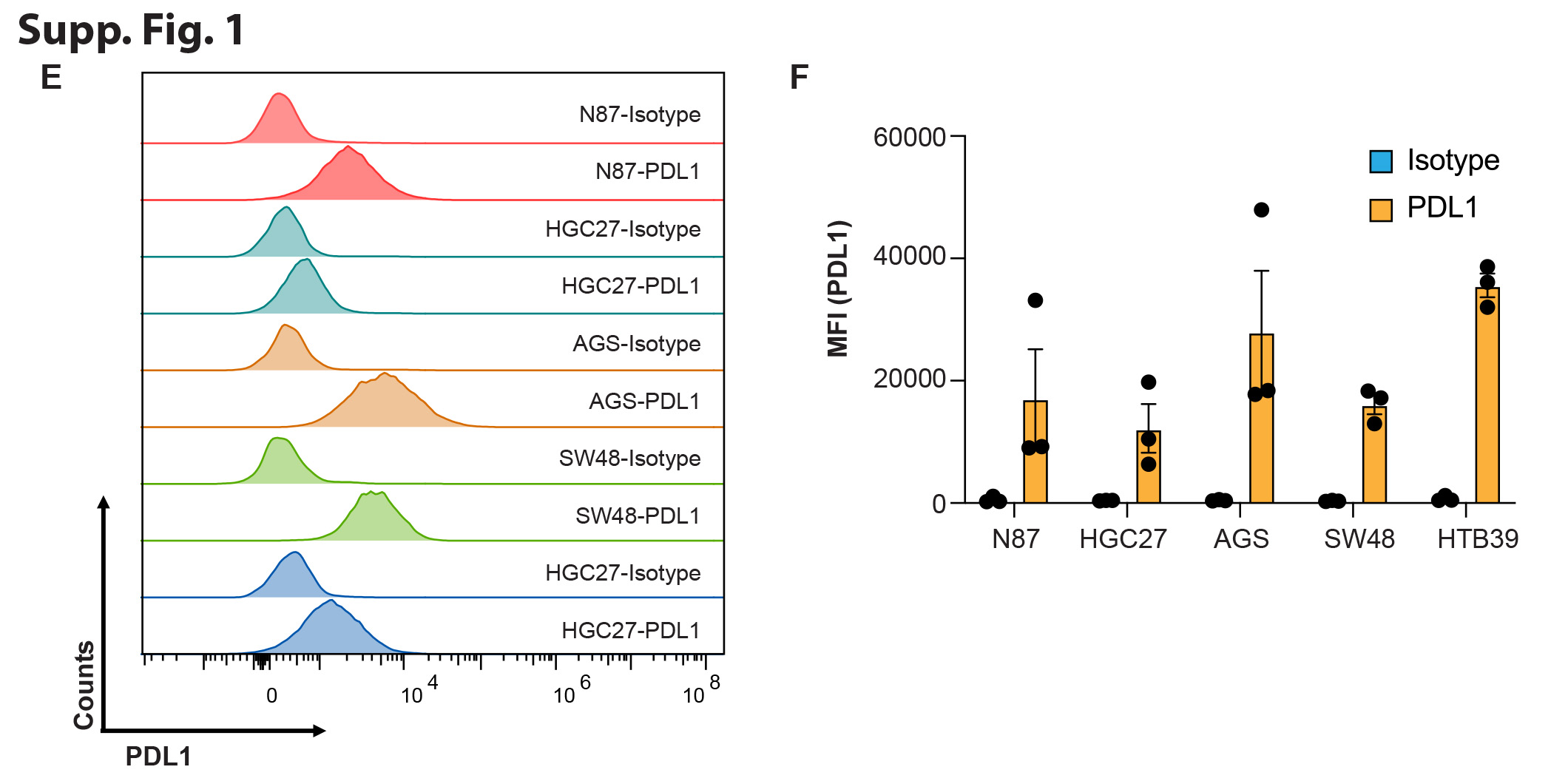

Supplement: Supplementary file 2 — Supporting information [file CTM2-14-e70057-s004.jpg]

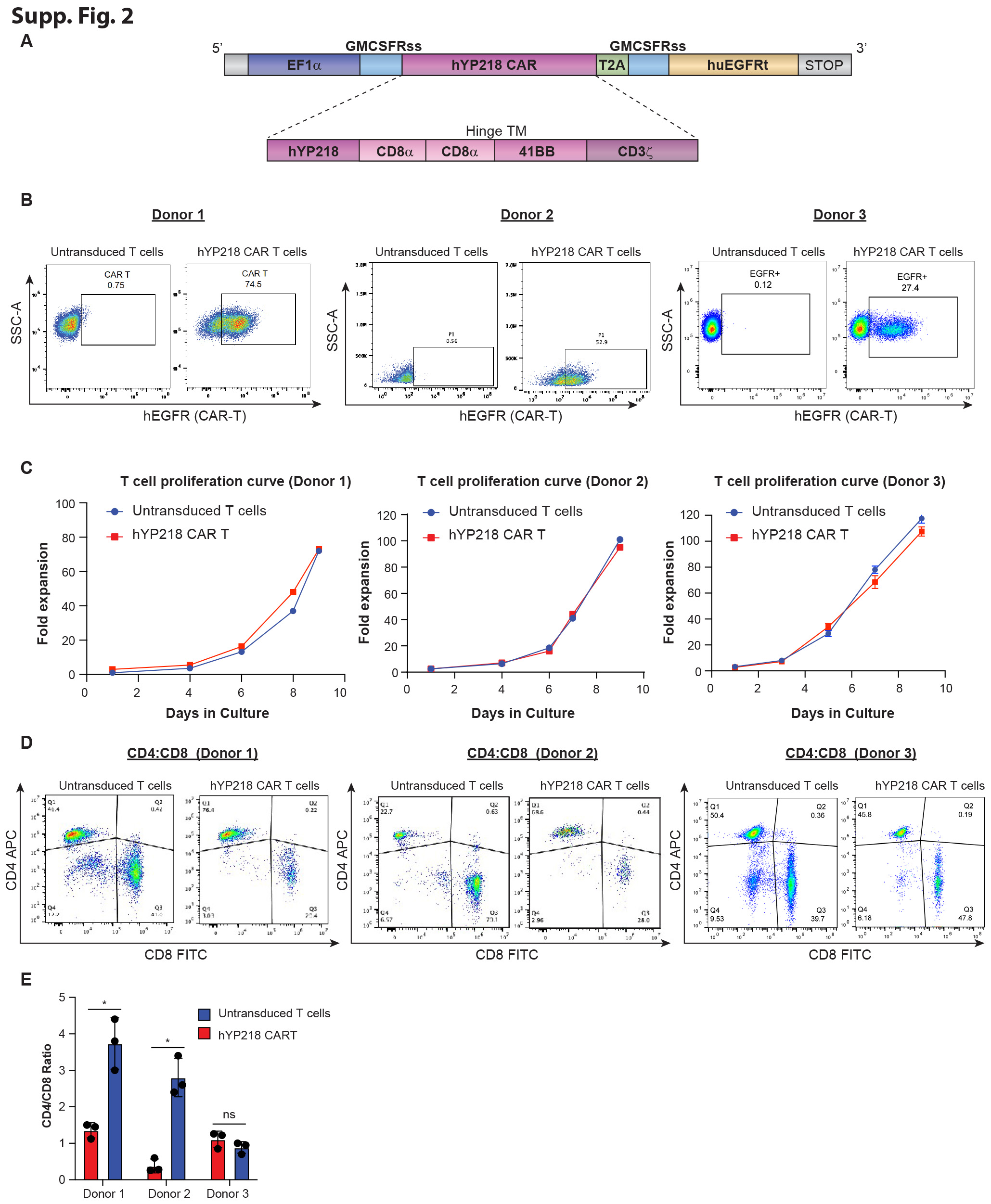

Supplement: Supplementary file 3 — Supporting information [file CTM2-14-e70057-s002.jpg]

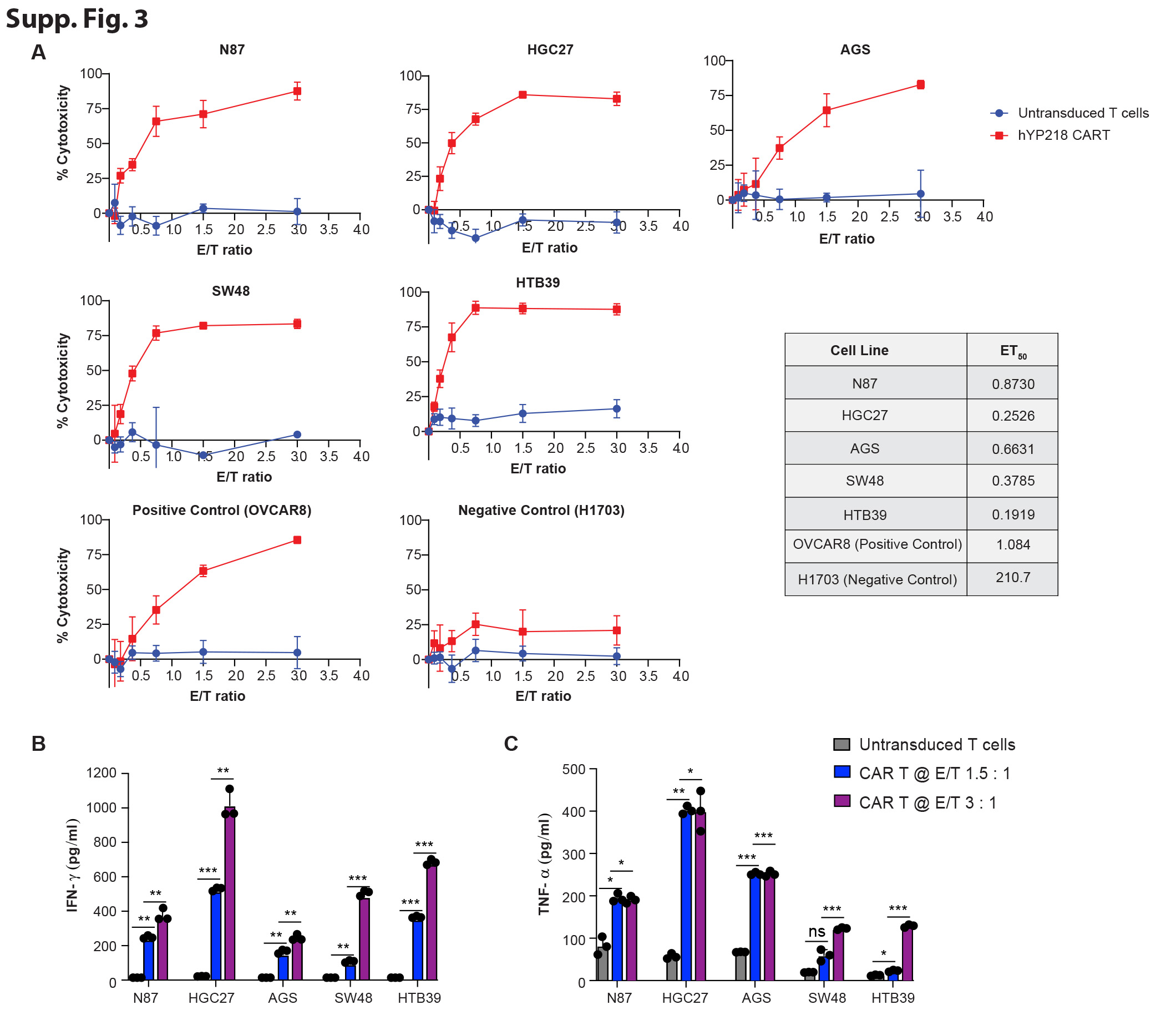

Supplement: Supplementary file 4 — Supporting information [file CTM2-14-e70057-s014.jpg]

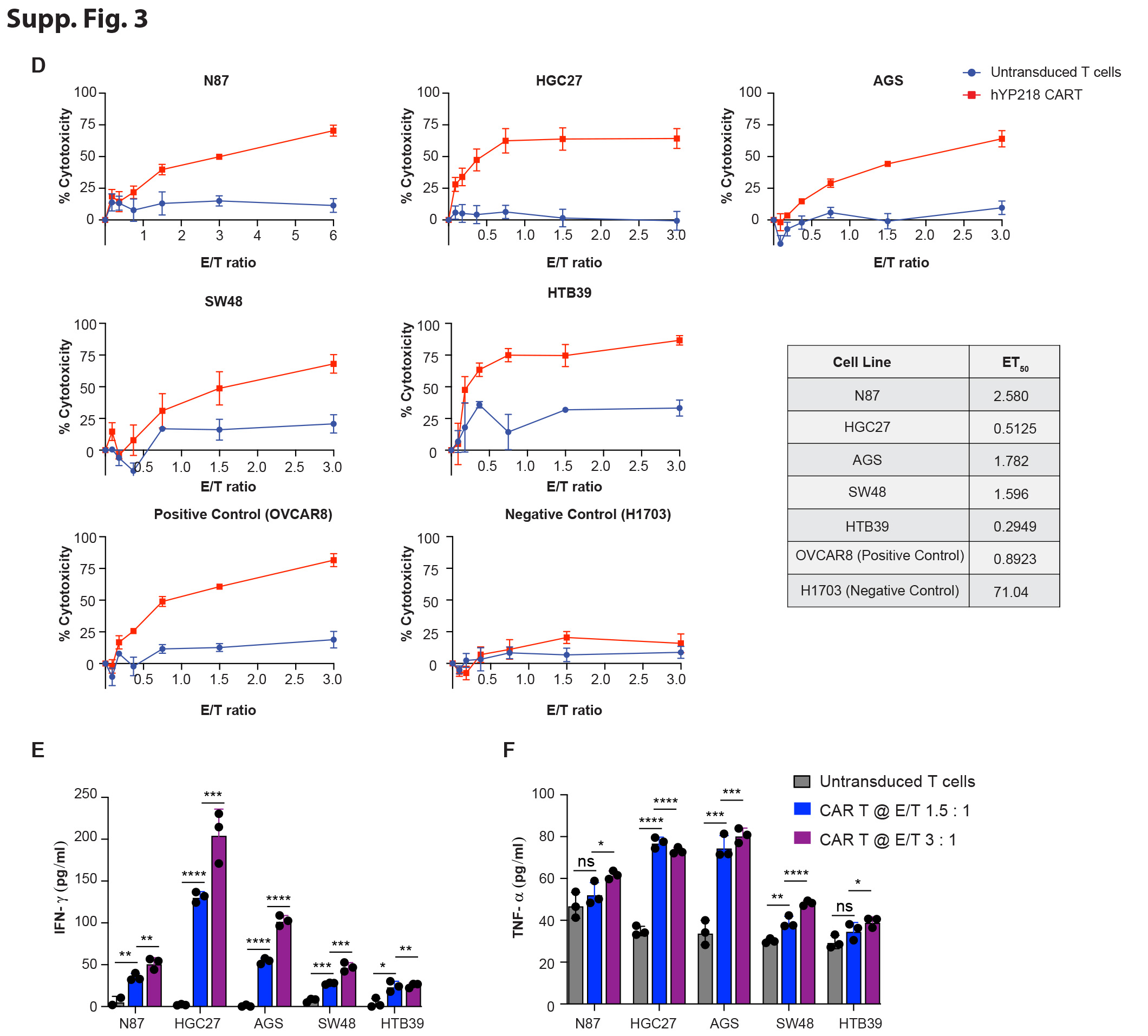

Supplement: Supplementary file 5 — Supporting information [file CTM2-14-e70057-s013.jpg]

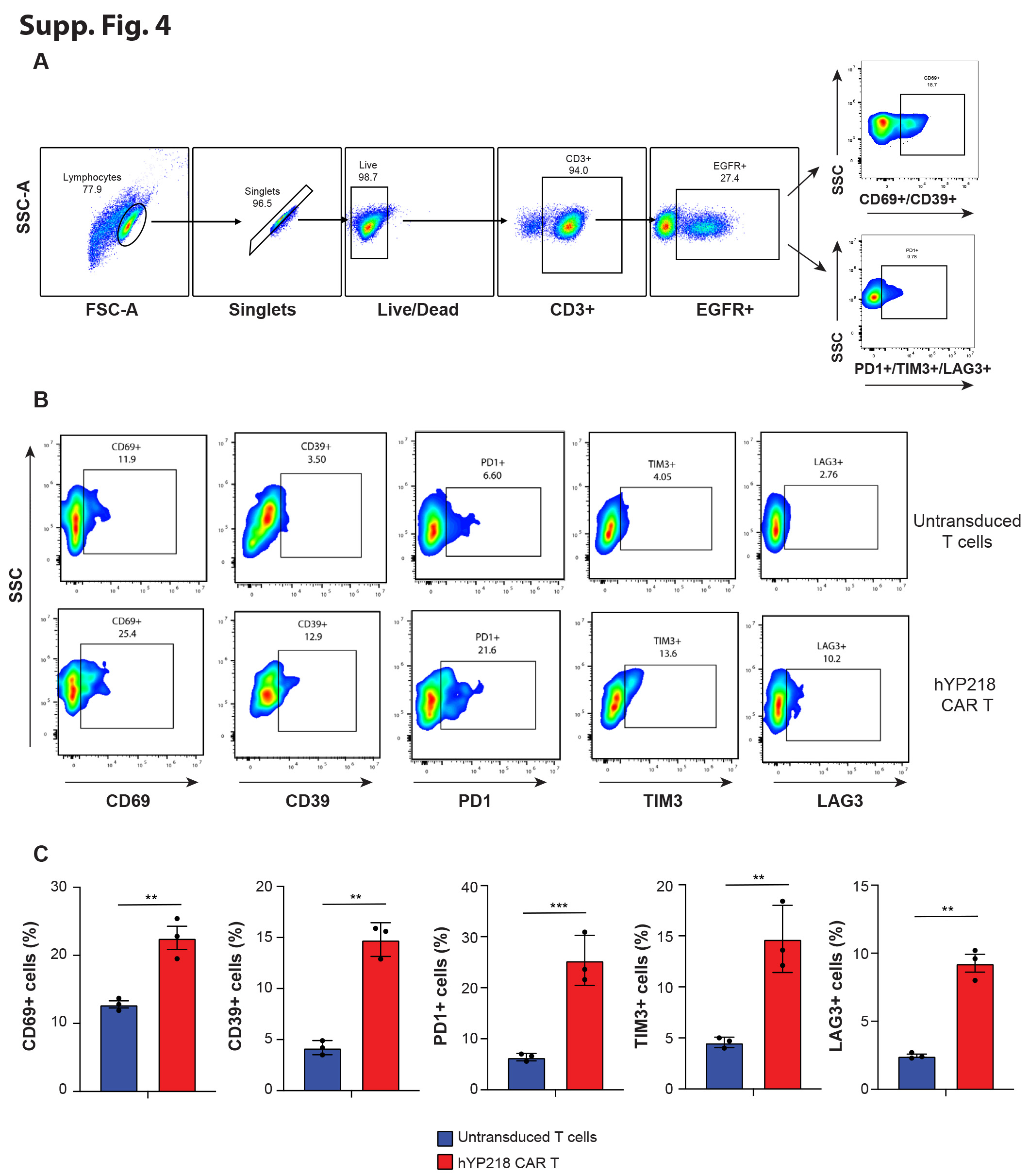

Supplement: Supplementary file 6 — Supporting information [file CTM2-14-e70057-s008.jpg]

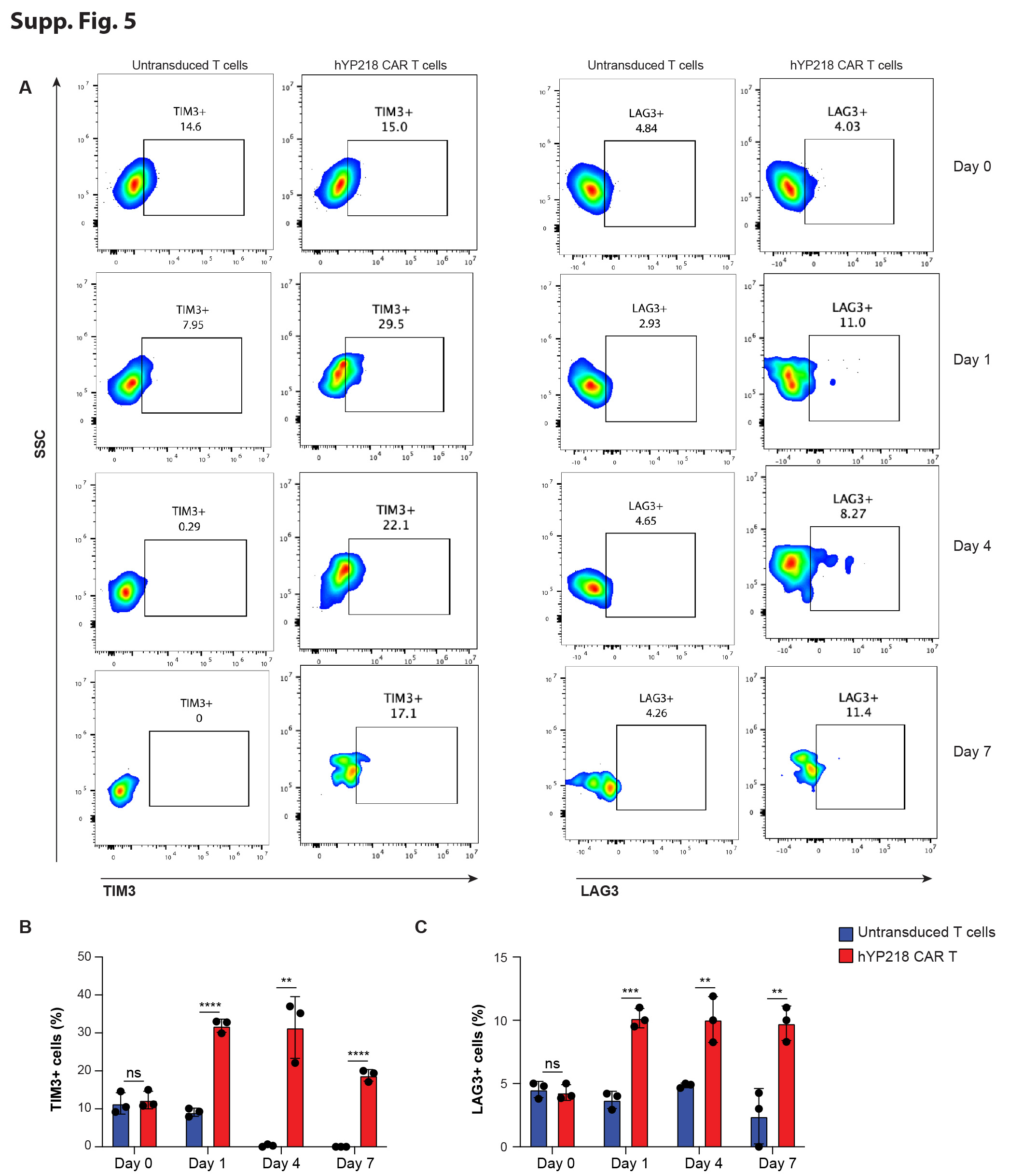

Supplement: Supplementary file 7 — Supporting information [file CTM2-14-e70057-s006.jpg]

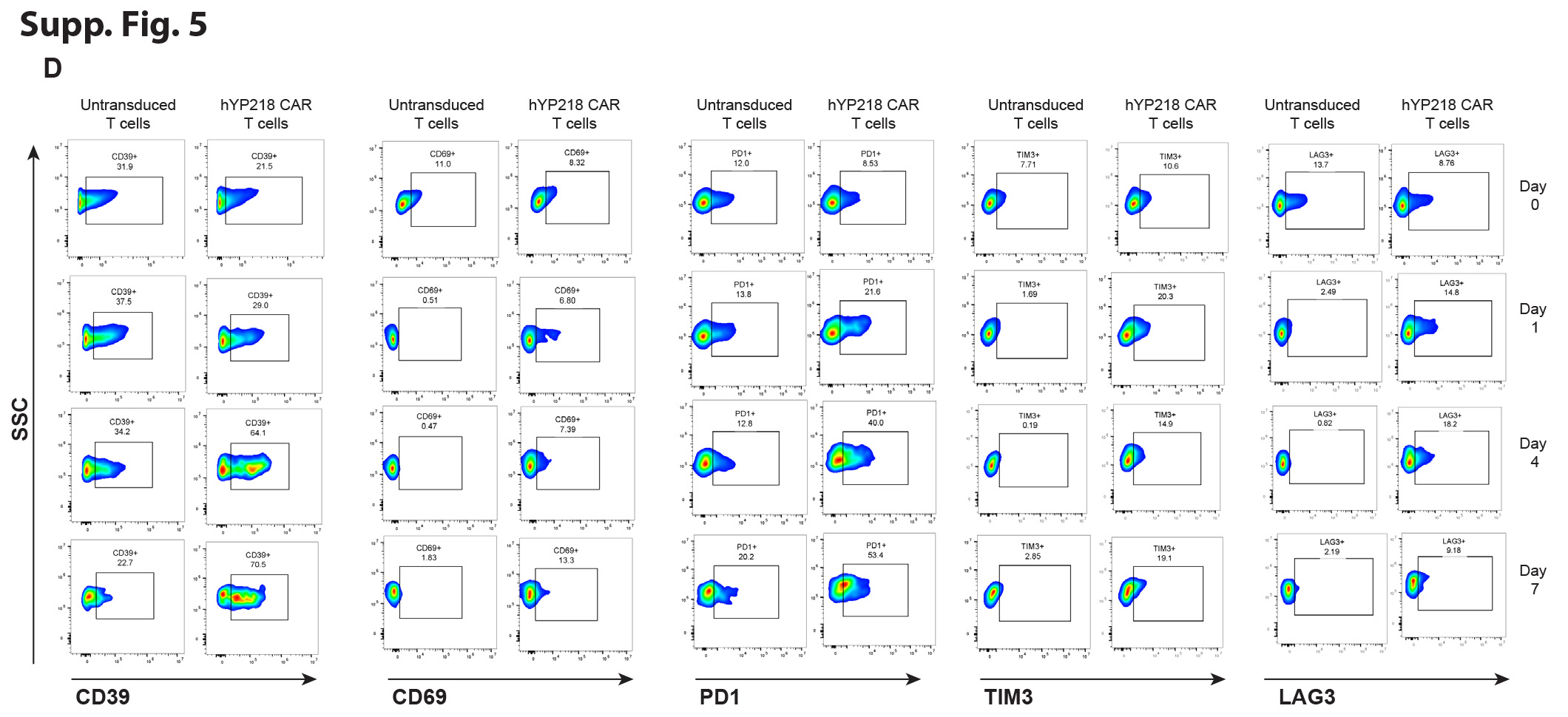

Supplement: Supplementary file 8 — Supporting information [file CTM2-14-e70057-s010.jpg]

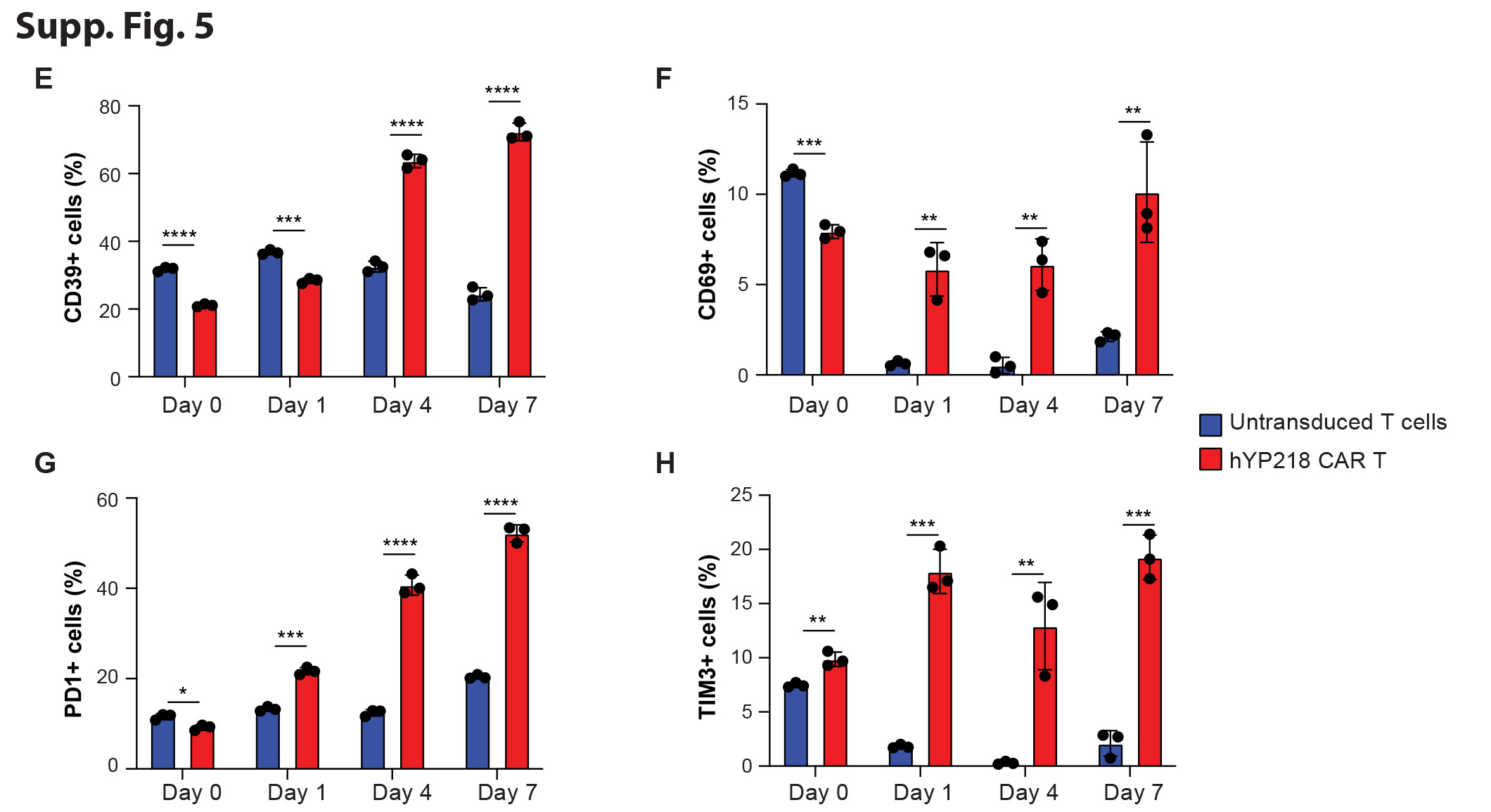

Supplement: Supplementary file 9 — Supporting information [file CTM2-14-e70057-s011.jpg]

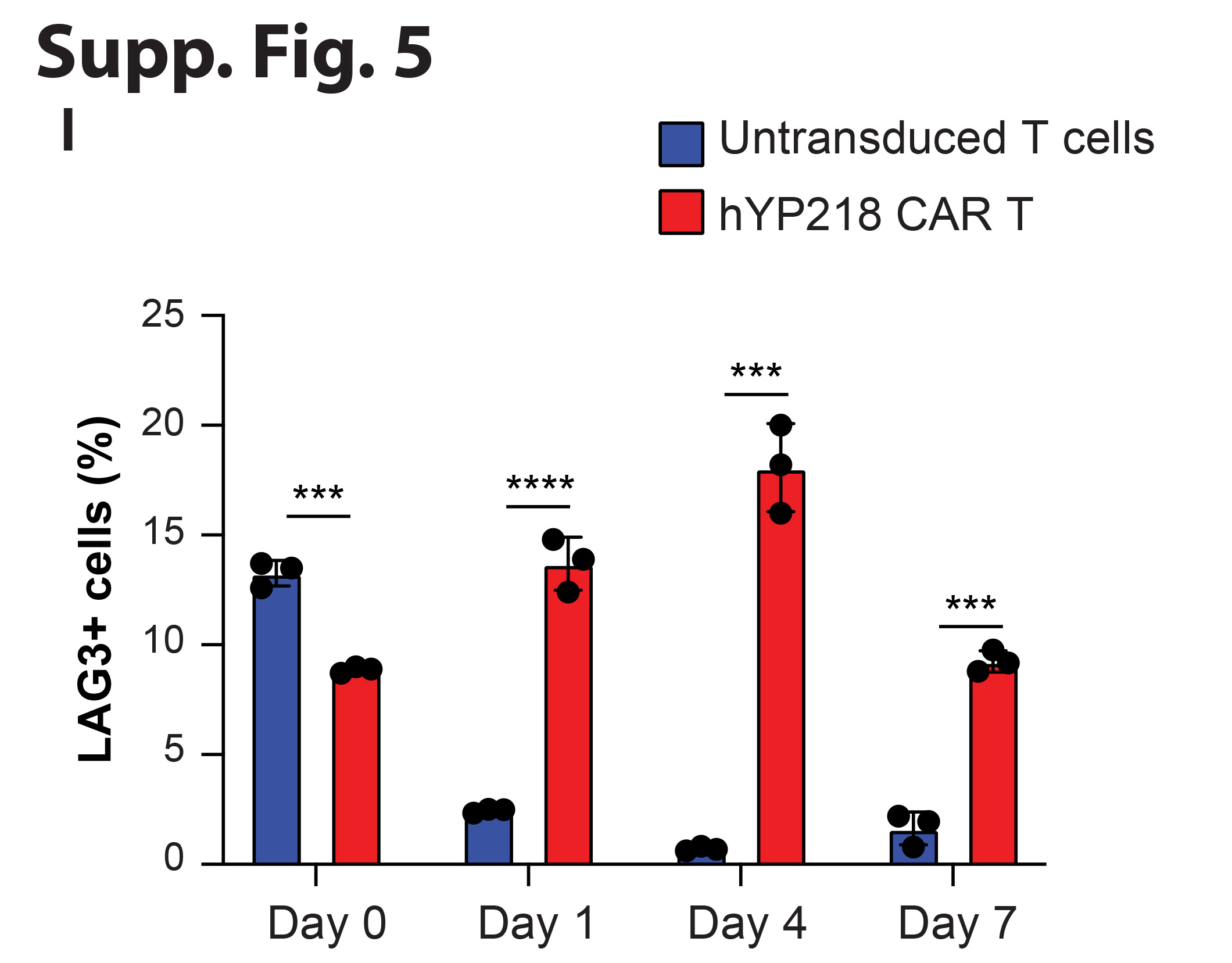

Supplement: Supplementary file 10 — Supporting information [file CTM2-14-e70057-s012.jpg]

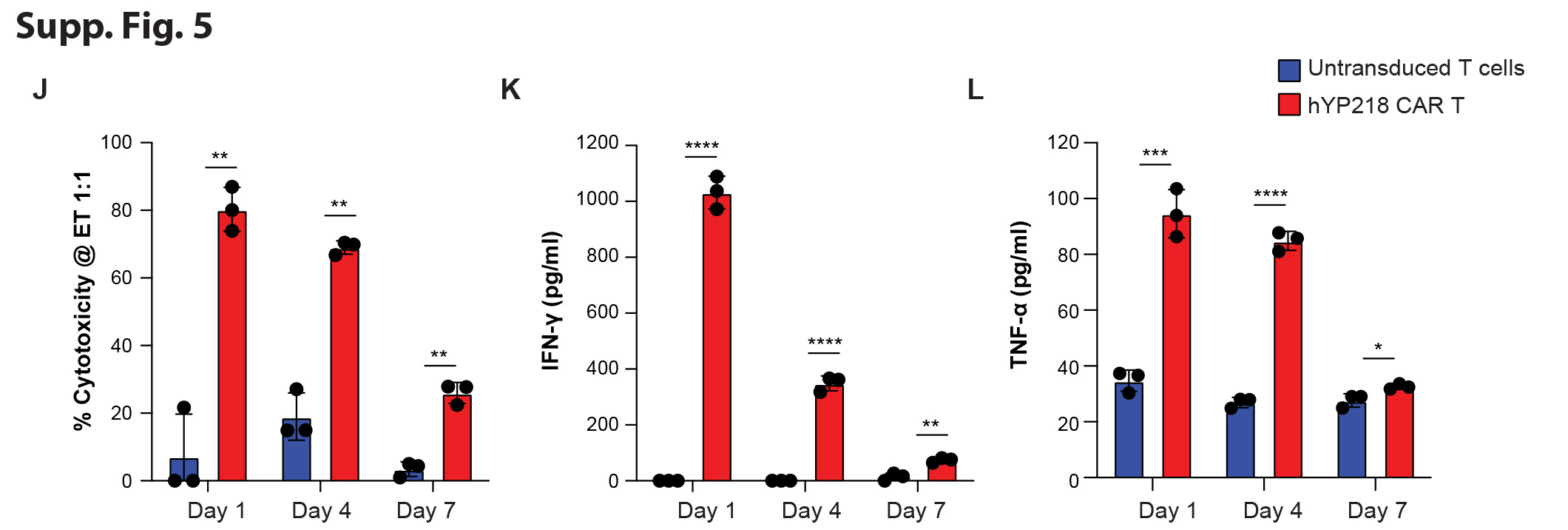

Supplement: Supplementary file 11 — Supporting information [file CTM2-14-e70057-s017.jpg]

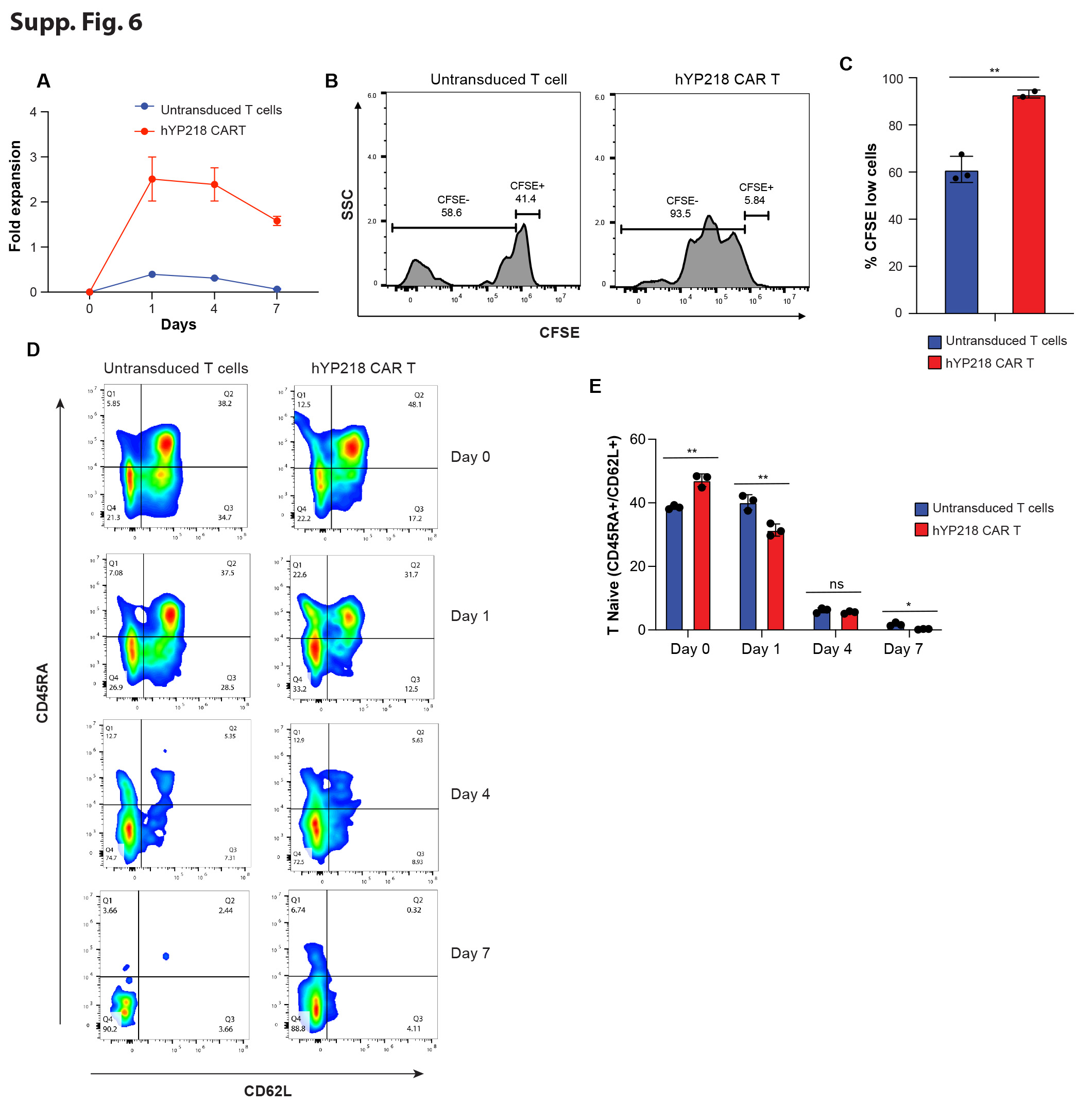

Supplement: Supplementary file 12 — Supporting information [file CTM2-14-e70057-s005.jpg]

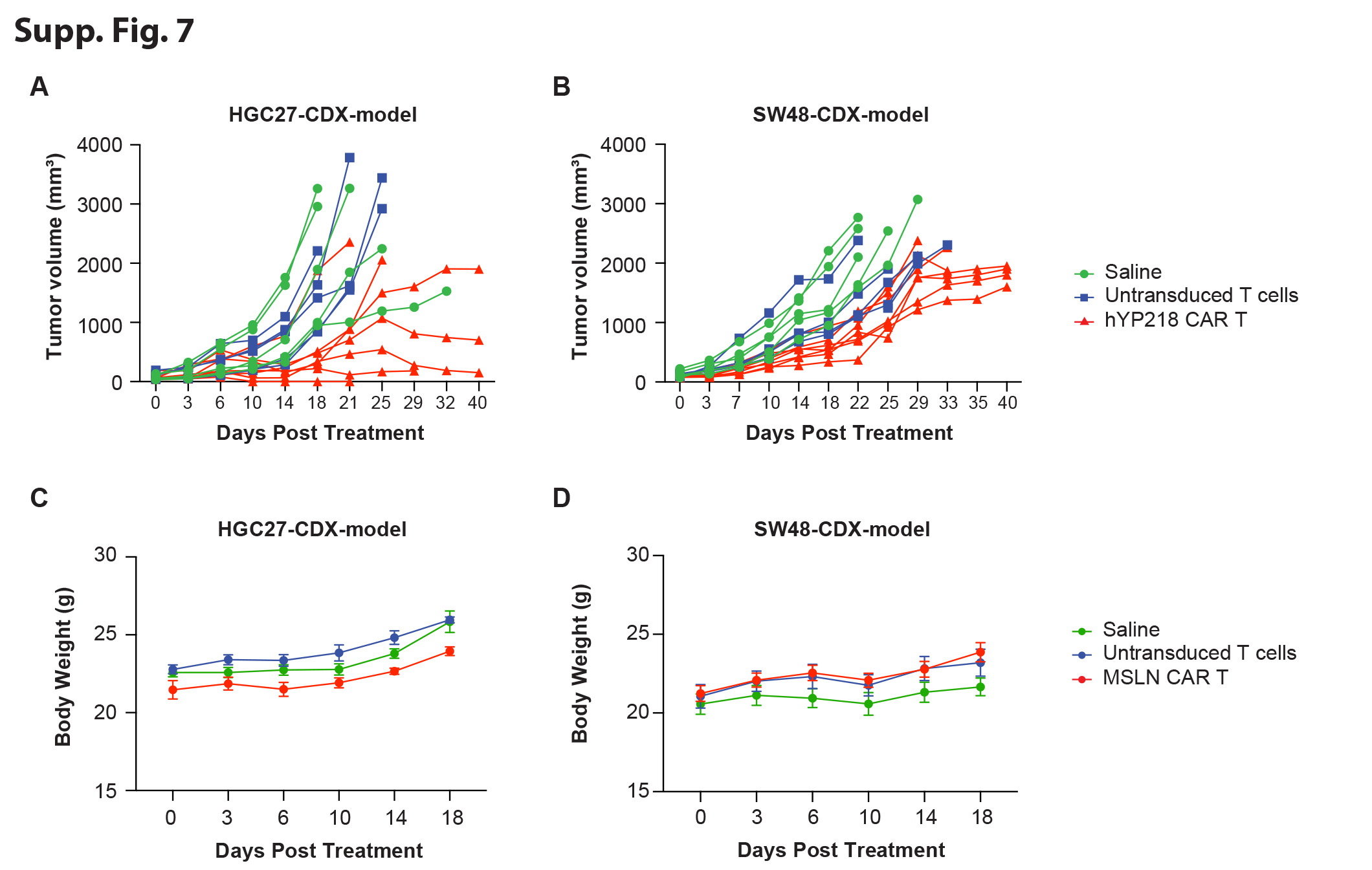

Supplement: Supplementary file 13 — Supporting information [file CTM2-14-e70057-s016.jpg]

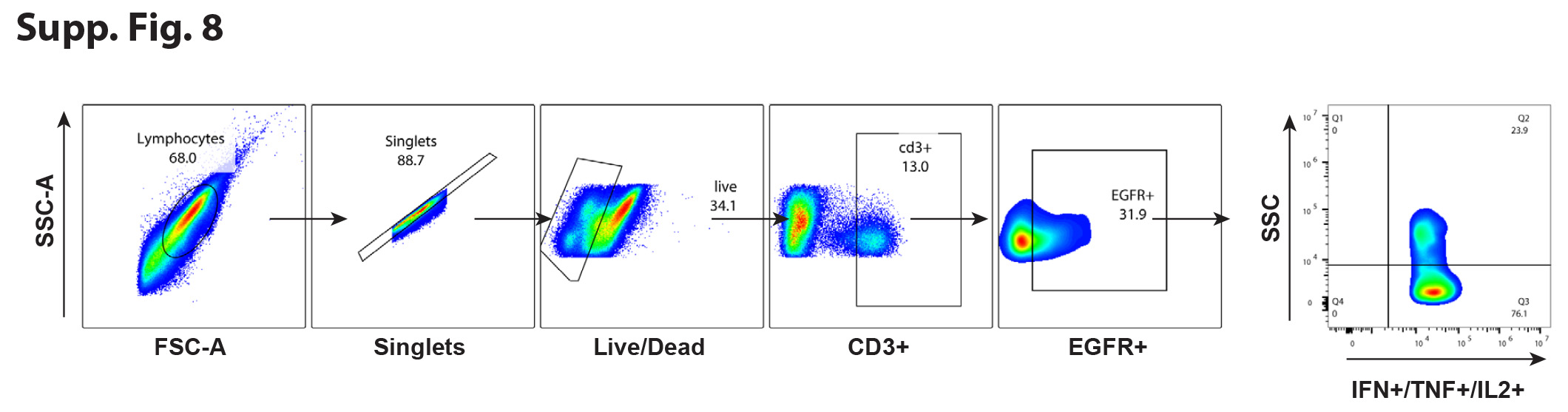

Supplement: Supplementary file 14 — Supporting information [file CTM2-14-e70057-s009.jpg]

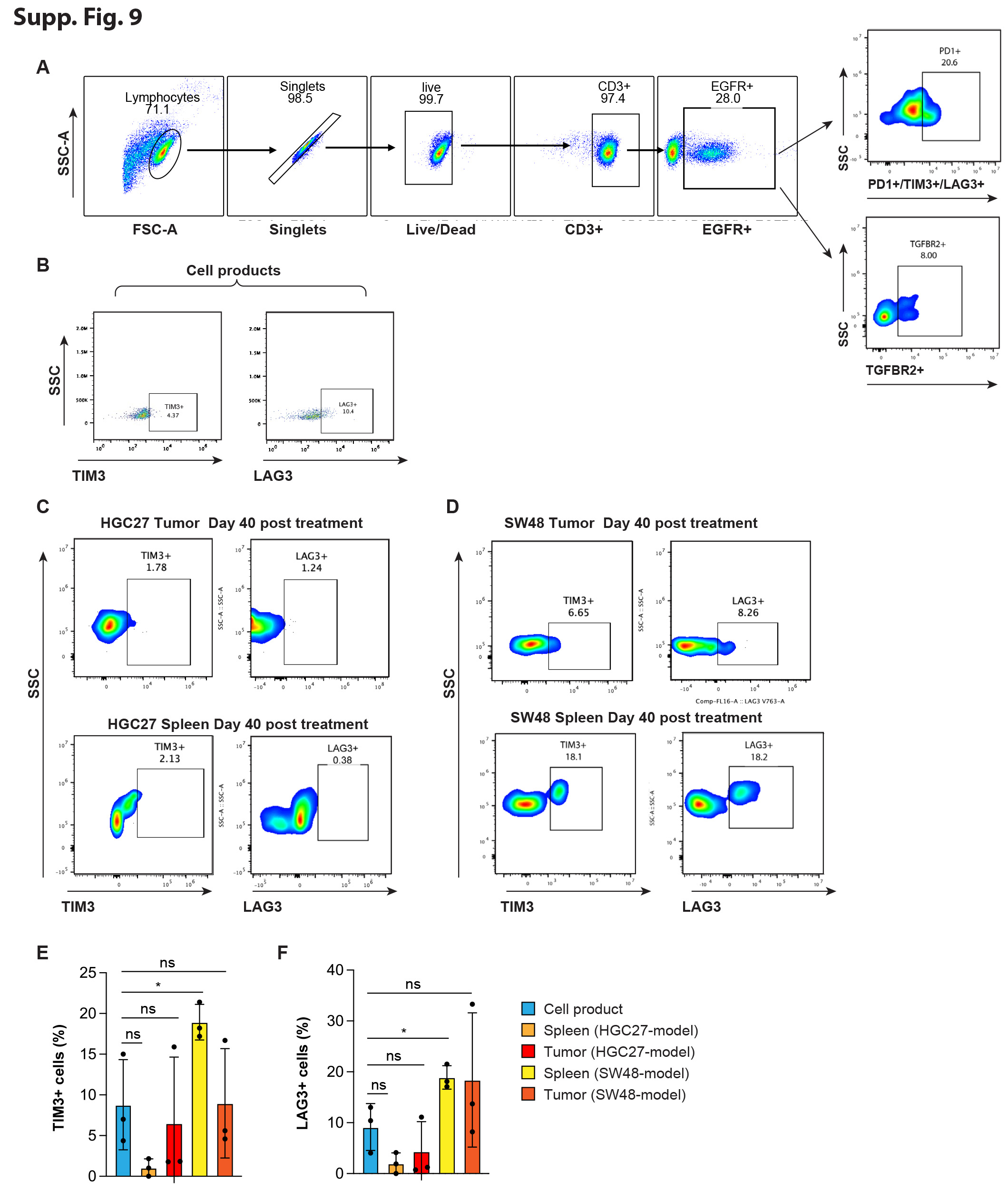

Supplement: Supplementary file 15 — Supporting information [file CTM2-14-e70057-s003.jpg]

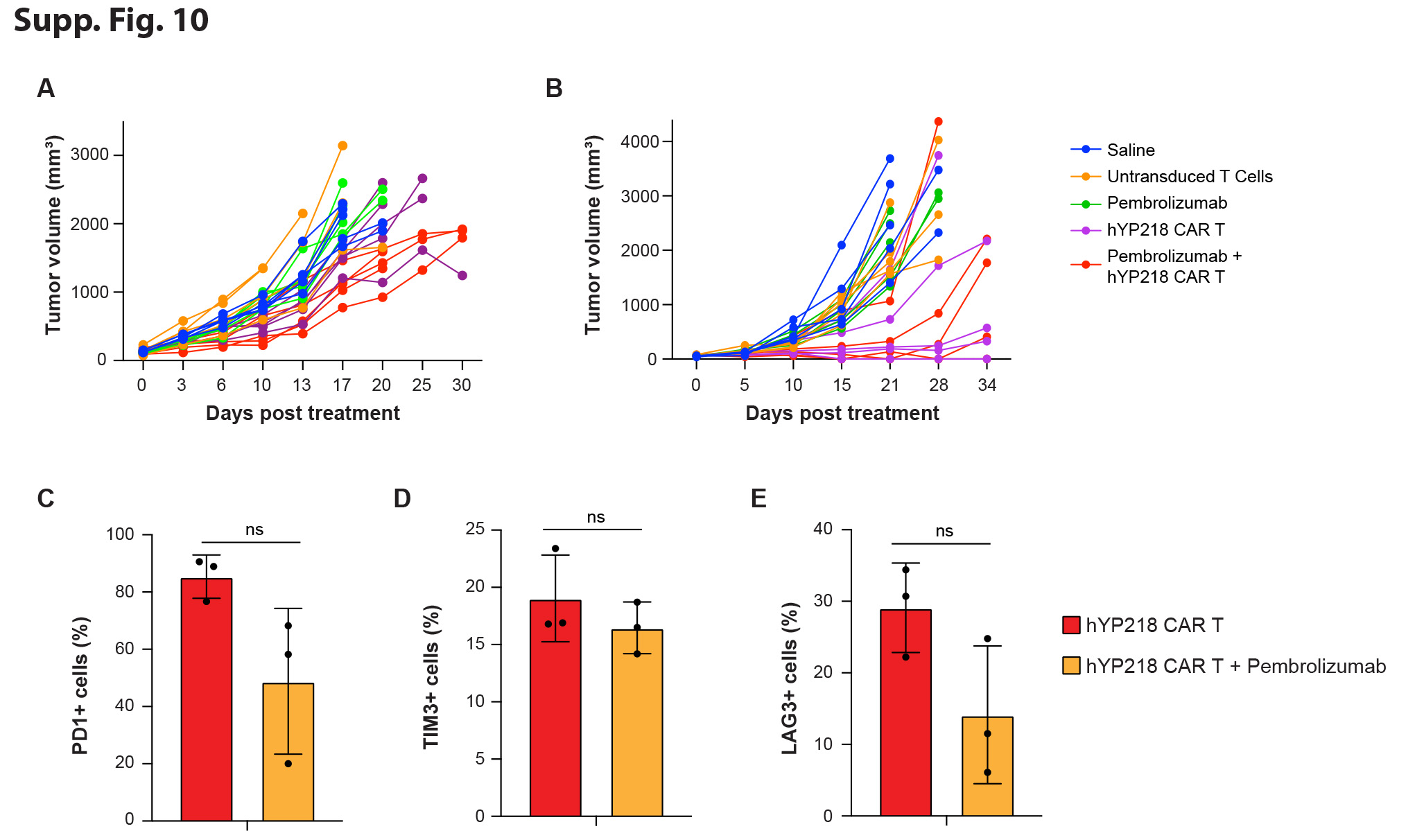

Supplement: Supplementary file 16 — Supporting information [file CTM2-14-e70057-s001.jpg]
