# Supplementary material for: Persistence of activated anti‐mesothelin hYP218 chimeric antigen receptor T cells in the tumour is associated with efficacy in gastric and colorectal carcinomas
Source: Clin Transl Med. 2024 Nov 15;14(11):e70057. doi: 10.1002/ctm2.70057 (PMC11567854; doi:10.1002/ctm2.70057)
Supplement: Supplementary file 17 — Supporting information [file CTM2-14-e70057-s007.docx]

**SUPPLEMENTARY FIGURE LEGENDS**

**Supplementary Fig. S1: (A)** MSLN expression in tumor biopsies and paired normal tissues of different cancer types based on analysis of data in the GEPIA (<http://gepia.cancer-pku.cn/>). See Supplementary table S1 for cancer types. T, tumor; N, normal tissue, n, number of samples used for analysis. (**B)** MSLN expression in gastric and colon carcinoma matched with TCGA normal and GTEx data. (**C-D)** Disease-free survival in patients with high and low levels of mesothelin expression for gastric and colorectal cancers. The analysis was performed using the MSLN expression data from the TCGA/GTEx datasets available within GEPIA. (**E-F)** PDL1 expression in various gastric and colorectal cancer cell lines. Data are plotted as mean ± SD (n=3; *P ≤ 0.05).

**Supplementary Fig. S2:**

**(A)** Schematic representation of CAR vector design.^28^ **(B)** Lentivirus transduction efficiency was assessed by measuring the percentage of CAR-expressing T cells after viral transduction of T cells obtained from two healthy donors (donor 1, donor 2 and donor 3). (C) T cell proliferation and expansion was evaluated by monitoring cell counts and fold expansion over time. (D-E) Flow cytometry analysis reveals the proportion of CD4+ and CD8+ T cell subsets within the CAR T cell population. Data represent mean ± SD (n=3). Significance levels: ns= non-significant; *P≤0.05.

**Supplementary Fig. S3:**

**(A)** Efficacy of cell killing by hYP218 CAR T cells derived from Donor-2 upon coculture with various cancer cell lines of gastric and colorectal carcinomas at different E:T ratios. E/T 50 ratio shown in the table. **(B-C**) Cytokine release profile depicting the production of IFN gamma and TNF alpha effector cytokines by hYP218 CAR T cells when cocultured with different cancer cell lines at E/T ratio of 1.5 and 3. Minimal to undetectable cytokine production was observed in the mock-treated groups, emphasizing the specific activation and reactivity of the hYP218 CAR T cells. (D-F) The efficacy of cell killing and the cytokine release profile by hYP218 CAR T cells derived from Donor-3 were assessed upon coculture with cancer cell lines. Data represent mean ± SD (n=3). Significance levels: ns= non-significant; *P≤0.05; **P≤0.01; ***P≤0.001; ****P≤0.0001.

**Supplementary Fig. S4:**

**(A)** Gating strategy for analyzing activation and exhaustion markers. (**B-C)** Evaluation of activation and exhaustion markers in hYP218 CAR T and untransduced T cells after 24 h of stimulation with HGC27 target cells. Data represent mean ± SD (n=3). Significance levels: **P≤0.01; ***P≤0.001.

**Supplementary Fig. S5**:

**(A-C)** Evaluation TIM3 and LAG3 exhaustion phenotypes in hYP218 CAR T cell repetitive killing assay with HGC27 cancer cells assay over 7 days. (**D-I)** Analysis of activation markers CD69 and CD39, and exhaustion markers PD1, TIM3, and LAG3 in hYP218 CAR T cells conducted in a repetitive coculture assay with MSLN+ SW48 cells. (**J-L)** Evaluation of cytotoxicity and cytokine release by hYP218 CAR T cells in a repetitive killing assay with SW48 cancer cells. Data represent mean ± SD (n=3). Significance levels: ns= non-significant; *P≤0.05; **P≤0.01; ***P≤0.001; ****P≤0.0001.

**Supplementary Fig. S6**:

**(A-C)** Fold expansion and CFSE staining of hYP218 CAR T and Untransduced T cells. (**D-E)** Analysis of T Naïve memory phenotype in hYP218 CAR T and Untransduced T cells. Data represent mean ± SD (n=3). Significance levels: ns= non-significant; *P≤0.05; **P≤0.01.

**Supplementary Fig. S7:**

**(A-B)** Tumor progression curves of individual mice for saline (n=5), untransduced T cells (n=5), and hYP218 CAR T (n=7) groups for HGC27 and SW48-CDX models, respectively, monitored until humane endpoints. (**C-D)** Body weight of mice for the saline, untransduced T cells, and hYP218 CAR T cells for the HGC27 and SW48-CDX models, respectively.

**Supplementary Fig. S8**: Gating strategy for evaluating the expression of effector cytokines in tumor-infiltrating hYP218 CAR T cells upon activation with leukocyte activation cocktail.

**Supplementary Fig. S9:**

**(A)** Gating Strategy for evaluating exhaustion and inhibitory molecules. (**B-F)** Flow cytometry analysis showing the expression of TIM3 and LAG3 in cell products and on hYP218 CAR T cells obtained from the tumors and spleens of mice bearing HGC27 and SW48 tumors. No significant increase in TIM3 and LAG3 expression was observed on hYP218 CAR T cells isolated from tissue samples obtained 40 days after treatment. Data represent mean ± SD (n=3). Significance levels: ns= non-significant; *P≤0.05.

**Supplementary Fig. S10:**

**(A-B)** HGC27 gastric tumor growth was monitored in individual NSG mice across different treatment groups, including saline, untransduced T cells, pembrolizumab, hYP218 CAR T cells, and combination therapy. The experiment was conducted with two groups: one group with larger initial tumor volumes **(A)** and another group with smaller initial tumor volumes at the start of treatment **(B)**. **C-E.** Evaluation of exhaustion markers in tumor-infiltrating lymphocytes from mice treated with hYP218 CAR T cells only and hYP218 in combination with pembrolizumab. Data represent mean ± SD. Significance levels: ns= non-significant.

**Supplementary Table 1**: List of abbreviations and corresponding names shown in figure 1A based on standard nomenclature as recognized by TCGA and as presented in the GEPIA tool.

| ACC | Adrenocortical carcinoma |
| --- | --- |
| BLCA | Bladder Urothelial Carcinoma |
| CESC | Cervical squamous cell carcinoma and endocervical adenocarcinoma |
| CHOL | Cholangio carcinoma |
| COAD | Colon adenocarcinoma |
| ESCA | Esophageal carcinoma |
| KIRC | Kidney renal clear cell carcinoma |
| KIRP | Kidney renal papillary cell carcinoma |
| LIHC | Liver hepatocellular carcinoma |
| LUAD | Lung adenocarcinoma |
| MESO | Mesothelioma |
| OV | Ovarian serous cystadenocarcinoma |
| PAAD | Pancreatic adenocarcinoma |
| PRAD | Prostate adenocarcinoma |
| READ | Rectum adenocarcinoma |
| STAD | Stomach adenocarcinoma |
| UCEC | Uterine Corpus Endometrial Carcinoma |

**Supplementary Table 2**: List of antibodies used in this study.

| **Antibody** | **Manufacturer** | **Catalog no.** |
| --- | --- | --- |
| anti-Mesothelin | Rockland | 200-301-A88 |
| anti-EGFR | R & D systems | FAB 9577P |
| anti-CD3 | BD Biosciences | 566953 |
| anti-CD39 | Biolegend | 328206 |
| anti-CD69 | Biolegend | 310932 |
| anti-PD1 | Biolegend | 393606 |
| anti-TIM3 | Biolegend | 345030 |
| anti-LAG3 | Biolegend | 369322 |
| anti-CD4 | Biolegend | 300514 |
| anti-CD8 | Biolegend | 344703 |
| anti-TGFBR2 | R & D systems | FAB 241F |
| anti-IFNγ | Biolegend | 502506 |
| anti-TNFα | Biolegend | 502931 |
| anti-IL2 | Biolegend | 500321 |
| anti-CD3 (PE) | Biolegend | 300408 |
